# Exploring the Complexity of Considering Race in the Practice of Medicine

**DOI:** 10.15766/mep_2374-8265.11585

**Published:** 2026-03-17

**Authors:** Shawn Koh

**Affiliations:** 1 Associate Professor, Department of Medical Education, California University of Science and Medicine-School of Medicine

**Keywords:** Race-Conscious Medicine, Race-Based Medicine, Health Equity, Diversity, Equity, and Inclusion

## Abstract

**Introduction:**

In recent years, educators and clinicians have advocated moving away from race-based medicine toward race-conscious medicine. Yet, few studies have evaluated the impact of teaching preclerkship medical students skills to critically evaluate various frameworks for understanding the role of race in clinical decision-making. This educational innovation was designed to review various frameworks, prompt clinical evaluation, and evaluate how student perspectives were shaped by their learning.

**Methods:**

We delivered a 90-minute interactive session focused on recognizing, appraising, and considering alternatives to the framework of race-based medicine. Ninety-two first-year medical students attended this mandatory session. Student opinions on how race should be used in medicine were measured via pre- and postsession Likert-style surveys (response rates 57% and 48%).

**Results:**

While most students initially thought race was a helpful marker of genetically associated disease risk, postsession responses shifted significantly (*p* = .039) toward neutrality or disagreement with this perspective. The presession survey showed varying perspectives on the use of race-based calculators and treatment guidelines. Postsession, the cohort shifted toward seeing these uses of race as more harmful than beneficial, with statistically significant perspective shifts on the use of race in estimated glomerular filtration rate calculation (*p* = .009), atherosclerotic cardiovascular disease risk calculation (*p* = .011), and treatment guidelines for hypertension and heart failure (*p* = .010).

**Discussion:**

This single session presentation led to increased concern regarding the use of race-based tools and guidelines in medicine. It supports the value of clinically relevant discussions of race and medicine with preclerkship medical students.

## Educational Objectives

By the end of this activity, learners will be able to:

1. Recognize the ways race is used as a proxy for genetic determinants of health.
2. Evaluate the use of race as an indicator of disease risk or response to therapy.
3. Assess the risks and benefits of the ongoing practice of race-based medicine.
4. Describe evolving frameworks for considering race in medicine.

## Introduction

Physicians have historically been trained to consider race in the epidemiology, diagnosis, and treatment of disease. The implicit framework that race has biological implications for diagnosis and treatment has been called “race-based medicine.” Critics of this view remind us that race is a social construct and question its use as a biological determinant in medical education, clinical decision-making, and research. An evolving framework, “race-conscious medicine,” emphasizes structural and social determinants as the primary drivers of race-based health disparities.^1^

Educators have identified common pitfalls in teaching race and medicine, including linking racial groups with particular diseases, presenting racial differences in disease burden without including social and structural context, and teaching clinical guidelines that use racial categories for diagnosing and treating disease.^2^ They have called for evolving curricula that eliminate nonscientific discussions of race as a biological risk factor, and that include discussions of the social and structural causes of race-related health care disparities.^1,3–5^ Medical students have joined faculty in highlighting the importance of discussions that question race as a proxy for genetic ancestry.^6–8^ These calls for reform have led to multifaceted responses for curriculum redesign. Among the suggested reforms are guidelines for revising teaching cases to highlight individual circumstances and various determinants of health rather than racial stereotypes,^3^ and developing structured race-conscious curricula for clerkship medical students.^5^

However, there are few published reports on the impact of specific educational innovations addressing student perspectives on the relationship between race and medicine. One program enrolled first-year medical student volunteers in a 10-hour series of trainings related to race in medicine. Survey results showed that participants were more likely than nonparticipants to recognize how race impacts medical care.^9^ While helpful in showing the general impact of education on race and medicine, that program did not specifically address concerns about the race-based medicine framework. A recent workshop taught medical students a tool for appraising the use of race in the medical literature, and showed that students valued the quality of the presentation, teaching, and materials^10^; however, changes in attitudes as a result of the workshop were not measured. One module showed that medical students who were taught about the use of race in estimated glomerular filtration rate (eGFR) calculations learned to appreciate the difference between race and ancestry and the clinical implications of race-based eGFR calculation. Interestingly, no statistically significant differences in student perceptions on the problematic impact of including race in algorithms were observed, because preintervention concern was already high.^11^

This 90-minute educational innovation was developed both to teach first-year medical students various frameworks for considering race and medicine and to measure how this would shape their attitudes toward these frameworks. This curriculum incorporated elements of the Master Adaptive Learner model, including critically appraising source material, applying what is learned to real-world settings, and engaging in informed self-assessment. The latter integrates reflection with external feedback and perspectives.^12^ The session encouraged practical application and critical analysis of race-based medicine by providing students an opportunity to utilize clinical calculators and guidelines that align with this framework. Interactive presentation tools and small-group discussions were incorporated to facilitate reflection and engagement with external feedback and perspectives. The evaluation survey included statements related to the use of race in diagnosis and treatment to assess whether student attitudes toward this framework would change as a result of the session.

## Methods

### Participants and Setting

This session on the role of race in clinical decision-making was delivered by a single educator (Shawn Koh) to 92 first-year medical students during a required year-long course, the Health Systems and Professional Practice I course presented at the California University of Science and Medicine. The course, delivered through 32 weekly sessions, explores the domains of lifelong learning, health systems science, ethics and professionalism, and diversity, equity, and inclusion. The current session, titled Race and Medicine, was presented during the second half of the academic year, after students had already studied the renal system and concomitantly with their cardiovascular system course. This time frame was chosen to ensure that students would be familiar with relevant race-based calculators and guidelines, in particular those for eGFR, hypertension, hyperlipidemia, and heart failure, that are regularly managed in primary care.

Prior to this session, students had been exposed to sessions on curiosity and intellectual humility, cultural humility, both/and thinking, and structural racism. Intellectual humility was presented as a scientific approach to new knowledge that avoids overidentification with one's previously held beliefs, and instead embraces rethinking and discovery.^13^ The session on structural racism, presented by another professor, described ways that racism is embedded in systems like housing, education, economic policy, and health care, with an emphasis that intention is not required for systems to cause harm. While the phrase “race-based medicine” was mentioned during the presentation, it was neither defined nor described in detail.

Students met in a large classroom, seated at tables with about 4–8 peers. The didactic portions of the presentation were delivered in several 5–15-minute blocks, each followed by a 5–10-minute period of small-group table discussions to promote reflection and intellectual curiosity (Appendix A). During these small-group discussions, the ∼12 faculty present were encouraged to join student groups and facilitate discussion without serving as content experts. In addition to receiving the assigned article and discussion questions beforehand, faculty were specifically briefed to model intellectual humility and avoid sharing their own opinions. This was done to promote psychological safety for reflection on varying perspectives. This instruction aligns with recommendations that sessions on cultural humility center around open discussions that avoid arriving at purportedly “correct” answers.^14^ Appendix B outlines the session format, time line, and detailed facilitator instructions.

### Implementation

Prior to the session, students were asked to read an article comparing race-based medicine with race-conscious medicine.^1^ This article was chosen because it included a table that summarizes frequently used race-based medicine tools, potential harmful impacts of these tools, and race-conscious alternatives for each. At the start of the session, students were invited to participate in a presession survey (Appendix C) using a QR code presented on several screens throughout the classroom.

#### Race as proxy for genetics

During the first section, we reviewed the US Census Bureau description of race, highlighting it as a social construct without claims of genetic significance. This was followed by a web-based interaction of student responses to this definition, which then transitioned to discussions on several ways race is used for risk-stratification, diagnosis, and treatment in medicine. We also explored how students are primed to do this through question stems in board-style questions.^15,16^ These examples were presented to promote student reflection on how well these uses of race squared with the current US Census Bureau description of race as a social construct. This section culminated with a first set of open-ended discussion questions on the benefits and risks or harms of using race in these ways.

#### Questioning race for risk, diagnosis, and therapy

A think-pair-share framework was then presented, describing the use of race to risk-stratify, diagnose, and treat patients. The intention was to highlight that this framework might link race with genetics. We followed this by presentation of an alternate framework that links race with geographic ancestry, pointing out evidence that contradicts that framework .^17^ This section concluded with several discussion questions on the link between race and biology.

#### Appraising race-based medicine

In this section, we discussed 3 common ways race is used in cardiovascular medicine: risk-stratification for cardiovascular disease risk and the use of statin medications, therapeutic options for first-line antihypertensive medications, and therapeutic options for heart failure. These examples were chosen to highlight the material they were studying for their cardiovascular system block. We then explored a case scenario where students were asked to make decisions about statin therapy and antihypertensive medication selection. This case required students to apply a real-world calculator and clinical guidelines for their decision-making. The intention was to help students see the connection between the theoretical framework of race-based medicine and the tangible impact on patient care. The case was designed so that formulaic use of the presented cardiovascular disease risk calculator would result in students recommending a statin if the patient was Black but not if the patient was White. Also, the number of first-line antihypertensive medication options would be limited for Black patients. Students then discussed the benefits and risks of using race in this manner.

#### Considering race-conscious medicine

In the final section, we discussed the elements of race-conscious medicine, defined as “an alternative approach that emphasizes racism, rather than race, as a key determinant of illness and health.”^1^ These discussions on race-conscious medicine were heavily based on an article by Cerdeña et al,^1^ as well as quotations from prominent articles that advocate for the exclusion of race entirely or the consideration of race from a social determinant of health perspective.^18–22^ One example presented was the recently developed PREVENT calculator for assessing cardiovascular disease risk. This race-free calculator includes a patient's zip code to estimate the social deprivation index for all patients, regardless of race.^20^ This final section concluded with discussion questions to appraise the framework of race-conscious medicine. Students then had the opportunity to complete the identical postsession survey (Appendix C) before leaving class.

### Evaluation

Students evaluated the session with pre- and postsession Likert-style surveys (Appendix C), which contained a total of 10 questions and used a 5-point Likert-style rating scale for responses (1 = *strongly disagree*, 5 = *strongly agree*). Survey questions explored students’ attitudes toward linking race with genetics and biology; the general consideration of race in diagnosis, therapy, and research; the use of race in specific formulas and clinical guidelines; and their confidence in their perspectives. The surveys were administered anonymously using Microsoft Forms. Surveys were not linked; therefore, chi-square analysis, performed using SPSS software version 28, was used to compare pre- and postsession responses. Evaluation of this educational innovation received approval by the Institutional Review Board of California University of Science and Medicine.

## Results

The session was delivered in March 2025 to 92 first-year medical students. Fifty-two students (57% response rate) completed the presession survey and 44 (48% response rate) completed the postsession survey. The Table shows the pre- and postsession Likert-style responses and the results of chi-square analysis comparing pre- and postsession responses for each question.

**Table.**
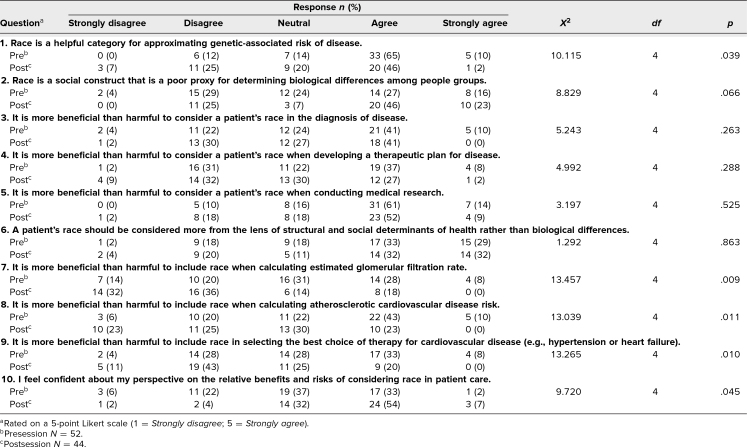
Pre- and Postsession Survey Responses Regarding First-Year Student Perspectives on the Use of Race in Clinical Decision-Making

Prior to the session, most students thought race was a helpful category for approximating genetically associated risk of disease (question 1). Their responses showed a significant shift (*p* = .039) away from this perspective after the session. In addition, students were more likely to disagree with statements linking race with biology, both before and after the session, when certain buzzwords like “social construct” and “structural and social determinants of health” were used (questions 2 and 6). When asked if consideration of race was more beneficial than harmful for each of the survey questions related to diagnosis (question 3), treatment (question 4), and research (question 5), student responses were widely spread and did not change significantly after the session. Their sustained response regarding medical research was particularly noteworthy, with both pre- and postsession attitudes leaning heavily in agreement that considering race is more beneficial than harmful in the conduct of medical research.

In contrast, when asked about the use of race for specific calculators and decision-making guidelines (questions 7, 8, and 9 on calculating eGFR, calculating atherosclerotic cardiovascular disease risk, and therapeutic choices for cardiovascular disease, respectively), student attitudes changed significantly as a result of the session, shifting toward agreement that considering race is more harmful than beneficial in these 3 areas (*p* = .009, *p* = .011, and *p* = .010, respectively). Finally, regardless of their perspectives, student confidence in their responses (question 10) increased (*p* = .045) after the session.

## Discussion

This educational innovation showed that first-year student perspectives on linking race with genetically associated disease risk were fairly diverse at baseline. Students were also receptive to material that promoted critical evaluation of this framework. Their engagement with the didactic presentation and small-group discussions led to increased concern with a race-based medicine framework. The curriculum has 3 strengths: inclusion of race-based formulas and guidelines to highlight clinical impact; utilization of learning strategies that promote intellectual humility; and encouragement of a nuanced understanding of race-conscious medicine.

Survey data also revealed that students’ attitudes on considering race in medicine varied based on how race is utilized. While the cohort shifted toward disagreeing with the use of race for specific formulas and guidelines (e.g., eGFR calculation, atherosclerotic cardiovascular disease risk estimation, choice of antihypertensive or heart failure medication), their attitudes regarding the general consideration of race in diagnosis and treatment were varied and did not change significantly after the session. Sharing specific guidelines and giving students the opportunity to try out risk assessment tools with case scenarios may be particularly effective in helping them to see the clinical relevance of various frameworks for race and medicine. Another reason specific calculators and guidelines were highlighted is that these have been the subjects of recent revision. The National Kidney Foundation and American Society of Nephrology recently published a recommendation for a revised calculation for eGFR that no longer considers race. This was done after extensive collaboration with experts, patients, providers, trainees, and community feedback and supported by social and scientific evidence.^23^ Similarly, the American Heart Association recently published updated 2025 hypertension guidelines that no longer consider race when choosing first-line pharmacologic therapy. In addition, the guidelines recommend an improved atherosclerotic cardiovascular disease risk calculator (known as PREVENT), validated by data from racially diverse US adults. This calculator does not include race. It instead utilizes zip code to estimate the social deprivation index.^24^ While the 2025 revised hypertension guidelines were not available when this session was delivered, they could be incorporated in future presentations on this topic to further promote critical evaluation of race-based medicine and provide a feasible way to consider social determinants of health.

This session was designed to promote rethinking and intellectual humility. While some presented materials challenged race-based medicine, additional materials and discussion questions left room for varying perspectives. Discussion segments were often concluded without delivering definitively correct answers. Alternative perspectives were presented to create space for various frameworks for race and medicine, rather than an either/or dichotomy. The intention behind this was 2-fold. First, as an educator who is a relative novice on this topic, the facilitator of this project (Shawn Koh) is still in the process of rethinking their own framework, and was hesitant to overstate this evolving perspective on race-based medicine. In addition, it was suspected that a presentation with a clear agenda might have been counterproductive to promoting open-mindedness and nuanced, both/and thinking. Such an approach could have potentially isolated students with dissenting views or led to defensiveness rather than curiosity. In hindsight, a downside to this approach is that some students may have left the session concluding that race-based medicine is an acceptable framework in health care. These reflections invite inquiry into whether a more impassioned approach might have been more effective, or perhaps less so, in promoting critical reflection. This could be explored by adding questions to the postsession survey on impact of the utilized methods on small-group discussions and the learning environment.

Students’ mixed perspectives on the general consideration of race for diagnosis and treatment (questions 3 and 4) may indicate a nuanced understanding of the association between race and health care outcomes. As presented in the session, a race-conscious medicine approach does not eliminate consideration of race entirely. Rather, it shifts the framework to one of structural and social determinants of health. Student variability in postsession perspectives on the risk/benefit ratio of generally considering race in diagnosis and therapy suggests their recognition of the distinction between questioning a formulaic consideration of race and excluding considerations of race altogether.

The evaluation of this educational innovation had several limitations. This pre- and postsession design was administered with immediate proximity to the session, limiting conclusions that can be drawn about more sustained attitude changes or lack thereof. It would be helpful to survey the same cohort of students later, during the clerkship phase of education. The evaluation was also limited by biases inherent to self-assessment. This may have been especially pronounced when buzzwords like “social construct” and “social determinants of health” were used. Students tended to disagree with linking race with biology in response to the relevant questions using these buzzwords (questions 2 and 6). The apparent discrepancy in response to these questions when compared to responses to the question in which such buzzwords were not used (question 1) should guide the wording of future surveys. The variation in responses to similar questions could also be a result of question ambiguity. To better understand student perspectives, future iterations could add questions with narrative responses. To increase the generalizability of results, this curriculum could be presented and evaluated at other institutions to see if student responses vary based on factors such as geography or the racial and ethnic demographics of various medical school localities. An additional limitation was the survey's focus on attitudes rather than behaviors. This could be addressed by conducting a similar educational innovation with clerkship students, residents, or attending physicians, with surveys exploring behaviors rather than attitudes.

This educational innovation supports the value of discussions of race in medicine as early as the preclerkship phase of undergraduate medical education. This curriculum could be adapted for various settings and participants. In a clinical setting with less allotted time for didactic teaching, a faculty member could share a case scenario involving race during rounds, asking trainees to discuss their decision-making process. The hypothetical patient's race could then be altered to prompt reflection on how this would impact their clinical reasoning. In contrast, a grand rounds–style presentation could reach a larger audience. Critical reflection could be facilitated by a panel of experts assigned to share the pros and cons of various frameworks for considering (or not considering) race in medicine. In both settings, especially with the presence of “experts” in the room, presenters should take care to avoid binary perspectives, and discussion should model cultural and intellectual humility.

In developing this innovation, the initial goal was to promote critical evaluation of the race-based medicine framework linking race with biology. Following session delivery and further reflection, it was recognized that alternative frameworks are much more nuanced than initially thought. For example, some experts still recommend the consideration of race when assessing disease disparities, albeit with a shift from biology to structural and social determinants.^1,22^ Others recommend not factoring race into assessment tools at all.^20,21^ It is also clear that guidelines and expert opinions are still evolving, with some experts currently only going as far as sharing ambivalence with the use of race-based medicine tools.^18,19^ As clinicians and educators, our understanding of the relationship between race and medicine is continually developing. This, however, should not deter us from teaching this topic to trainees. Rather, it presents us with the opportunity and responsibility to navigate this topic with nuance and critical reflection, modeling the humility and lifelong learning necessary to promote equitable systems of care.

## Appendices


Race and Medicine.pptxFacilitator Guide.docxPre- and Postsession Survey.docx
*All appendices are peer reviewed as integral parts of the Original Publication.*
