# Supplementary material for: Exploring the Complexity of Considering Race in the Practice of Medicine
Source: MedEdPORTAL. 2026 Mar 17;22:11585. doi: 10.15766/mep_2374-8265.11585 (PMC12992537; doi:10.15766/mep_2374-8265.11585)
Supplement: Supplementary file 1 — Race and Medicine.pptxFacilitator Guide.docxPre- and Postsession Survey.docx [file mep_2374-8265.11585-s001.zip › A. Race and Medicine.pptx]

## Slide 1
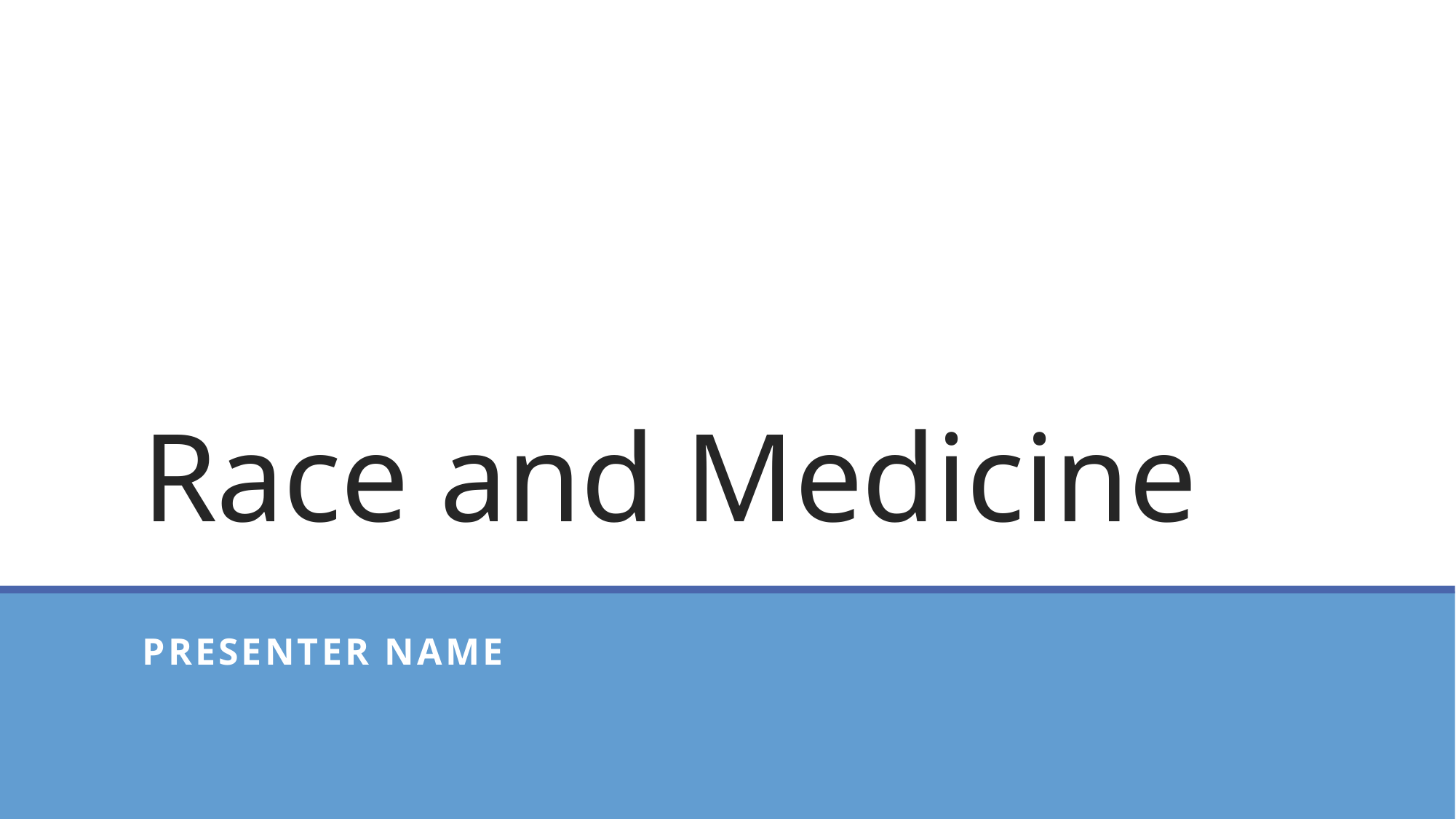

# Race and Medicine
Presenter Name

## Slide 2
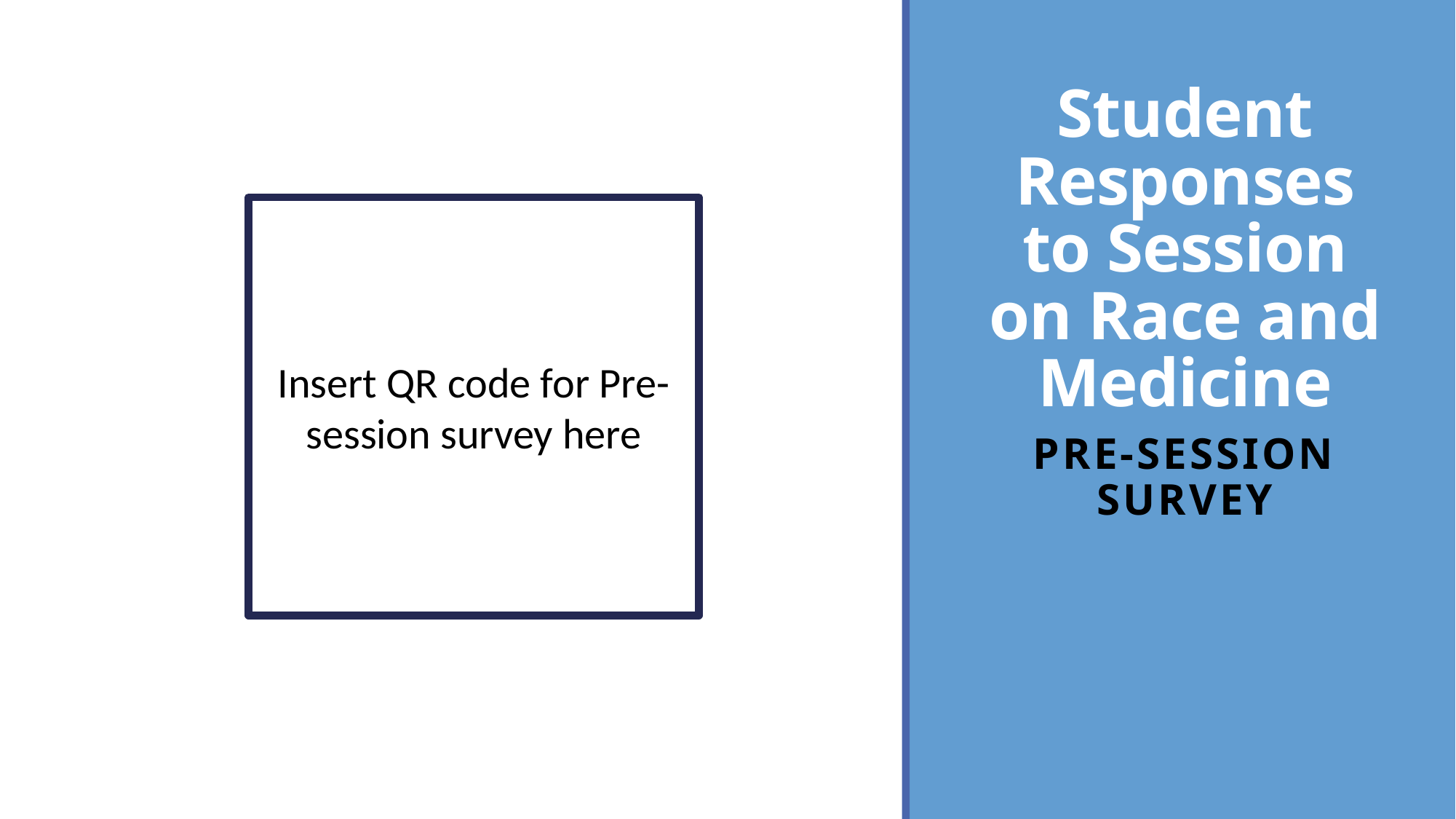

# Student Responses to Session on Race and Medicine
Insert QR code for Pre-session survey here
Pre-Session Survey

## Slide 3
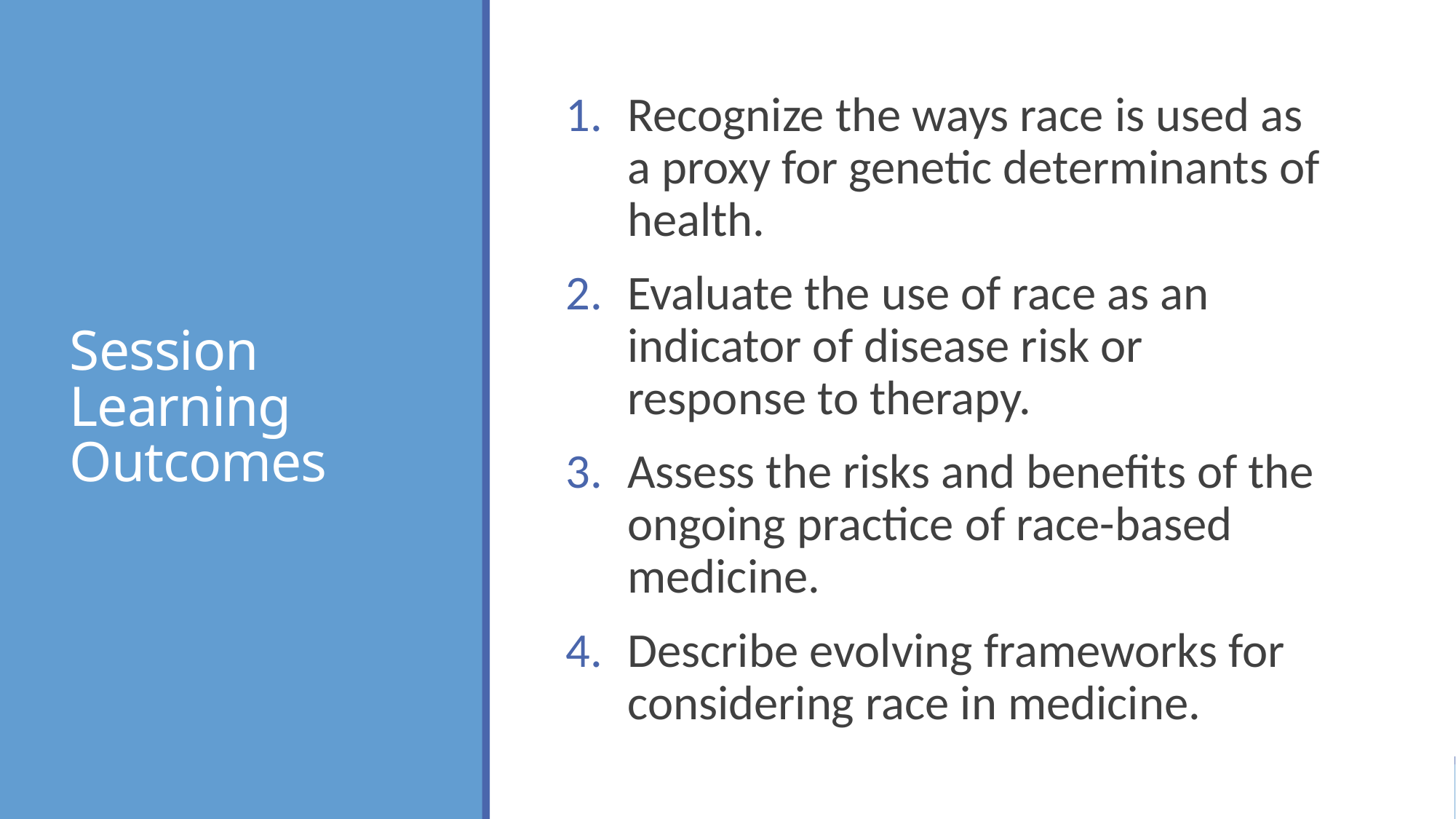

# Session Learning Outcomes
Recognize the ways race is used as a proxy for genetic determinants of health.
Evaluate the use of race as an indicator of disease risk or response to therapy.
Assess the risks and benefits of the ongoing practice of race-based medicine.
Describe evolving frameworks for considering race in medicine.

## Slide 4
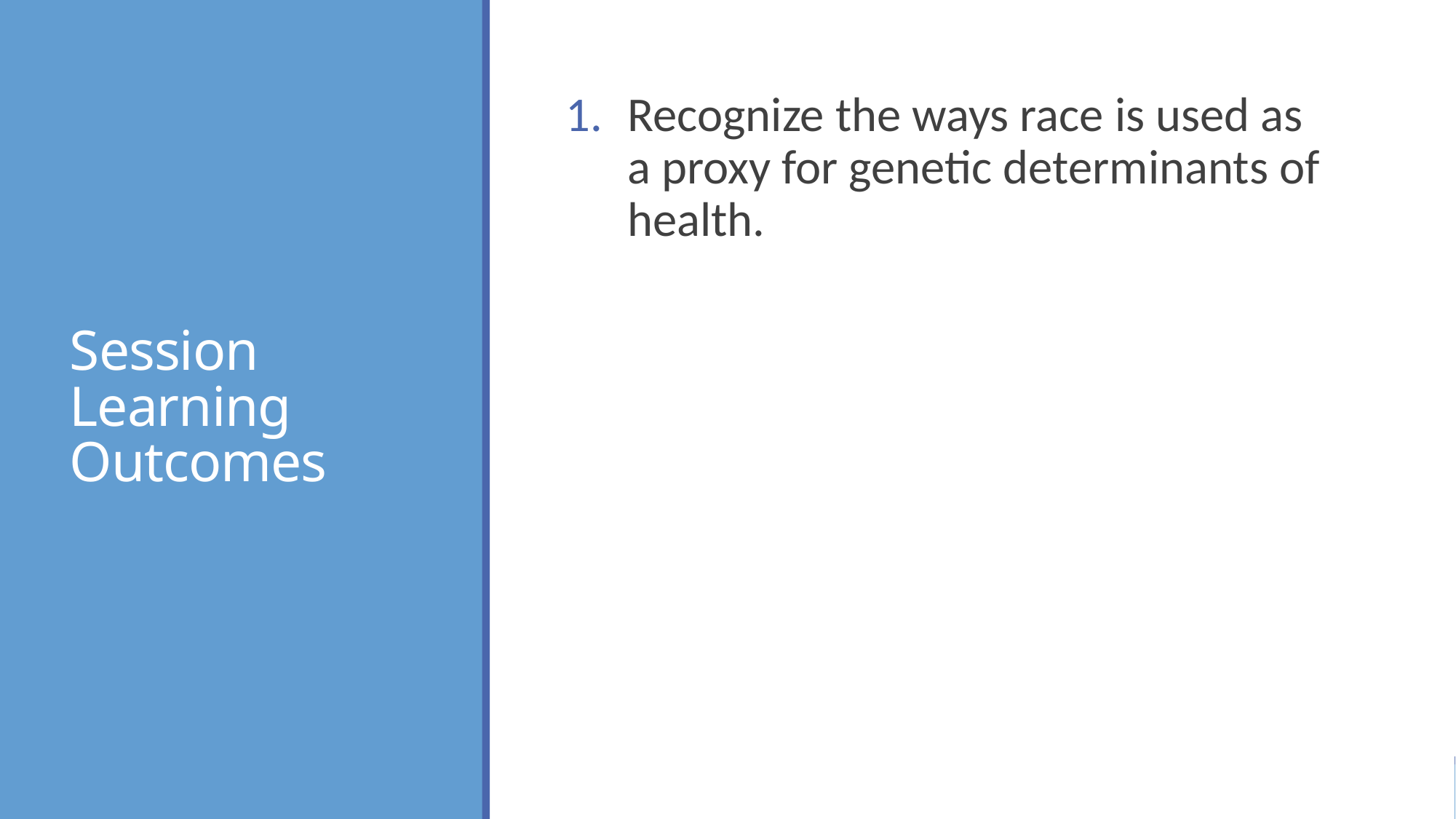

# Session Learning Outcomes
Recognize the ways race is used as a proxy for genetic determinants of health.
Evaluate the use of race as an indicator of disease risk or response to therapy.
Assess the risks and benefits of the ongoing practice of race-based medicine.
Describe evolving frameworks for considering race in medicine.

## Slide 5
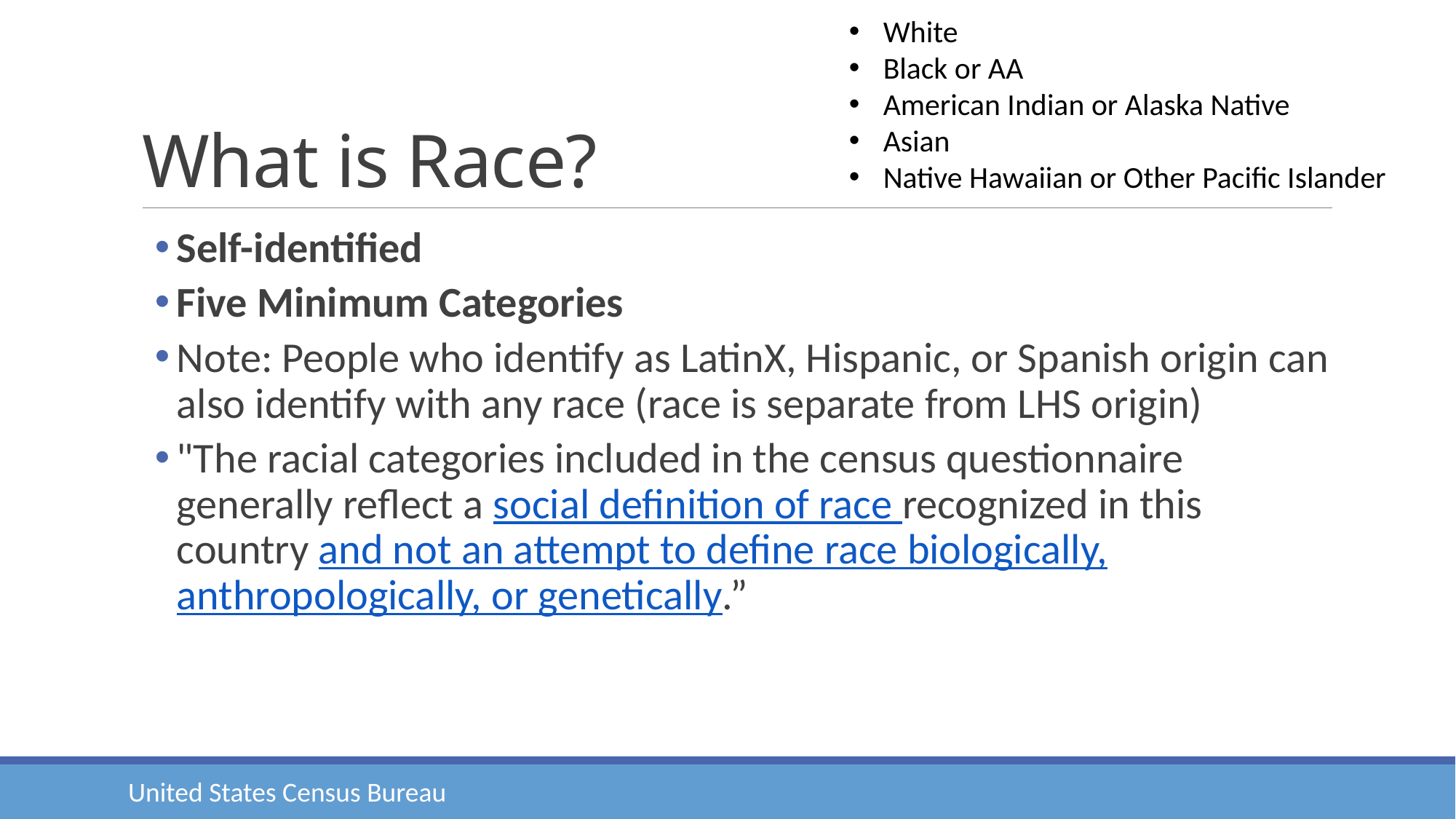

White
Black or AA
American Indian or Alaska Native
Asian
Native Hawaiian or Other Pacific Islander
# What is Race?
Self-identified
Five Minimum Categories
Note: People who identify as LatinX, Hispanic, or Spanish origin can also identify with any race (race is separate from LHS origin)
"The racial categories included in the census questionnaire generally reflect a social definition of race recognized in this country and not an attempt to define race biologically, anthropologically, or genetically.”
United States Census Bureau

## Slide 6
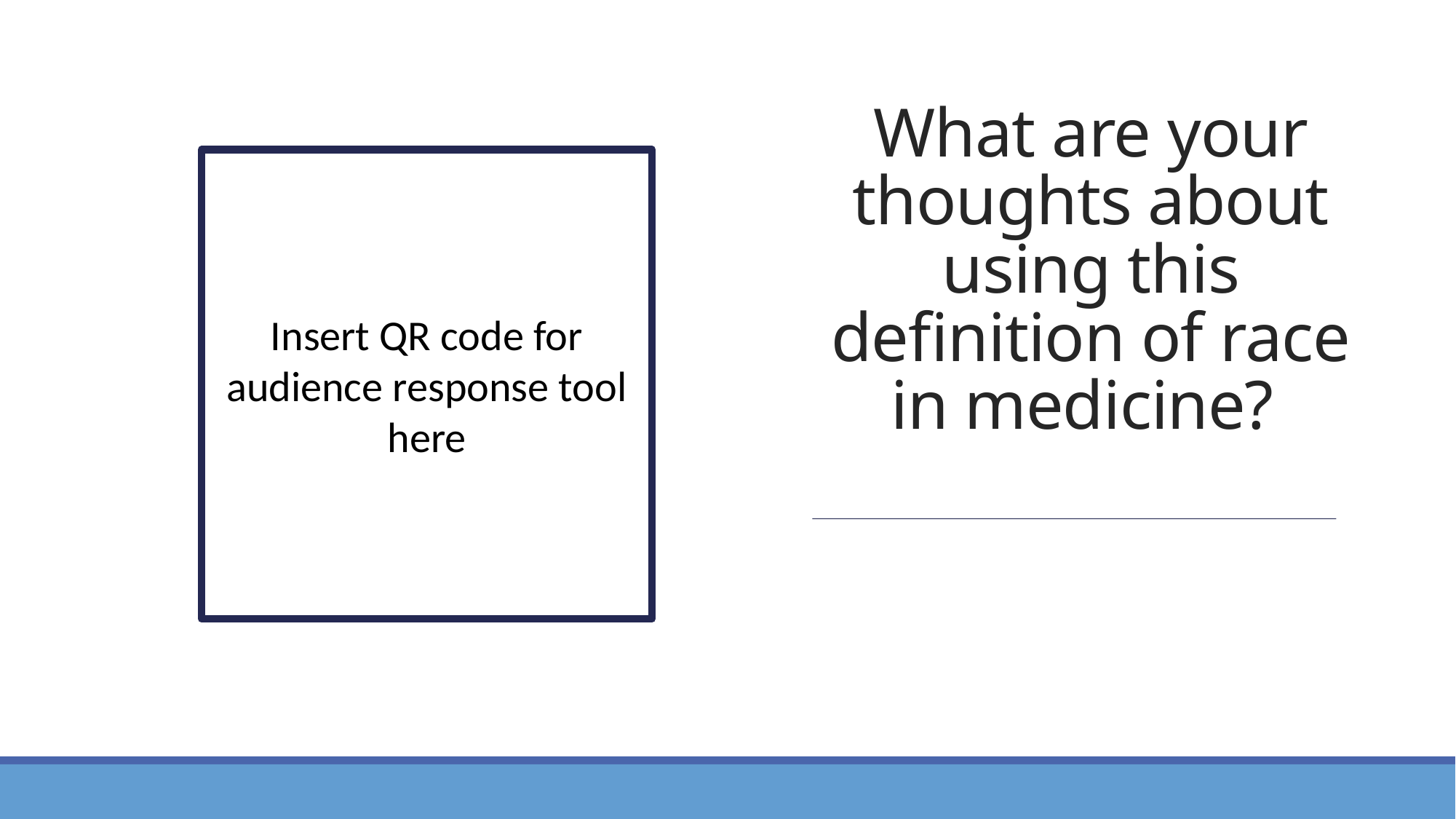

# What are your thoughts about using this definition of race in medicine?
Insert QR code for audience response tool here

## Slide 7
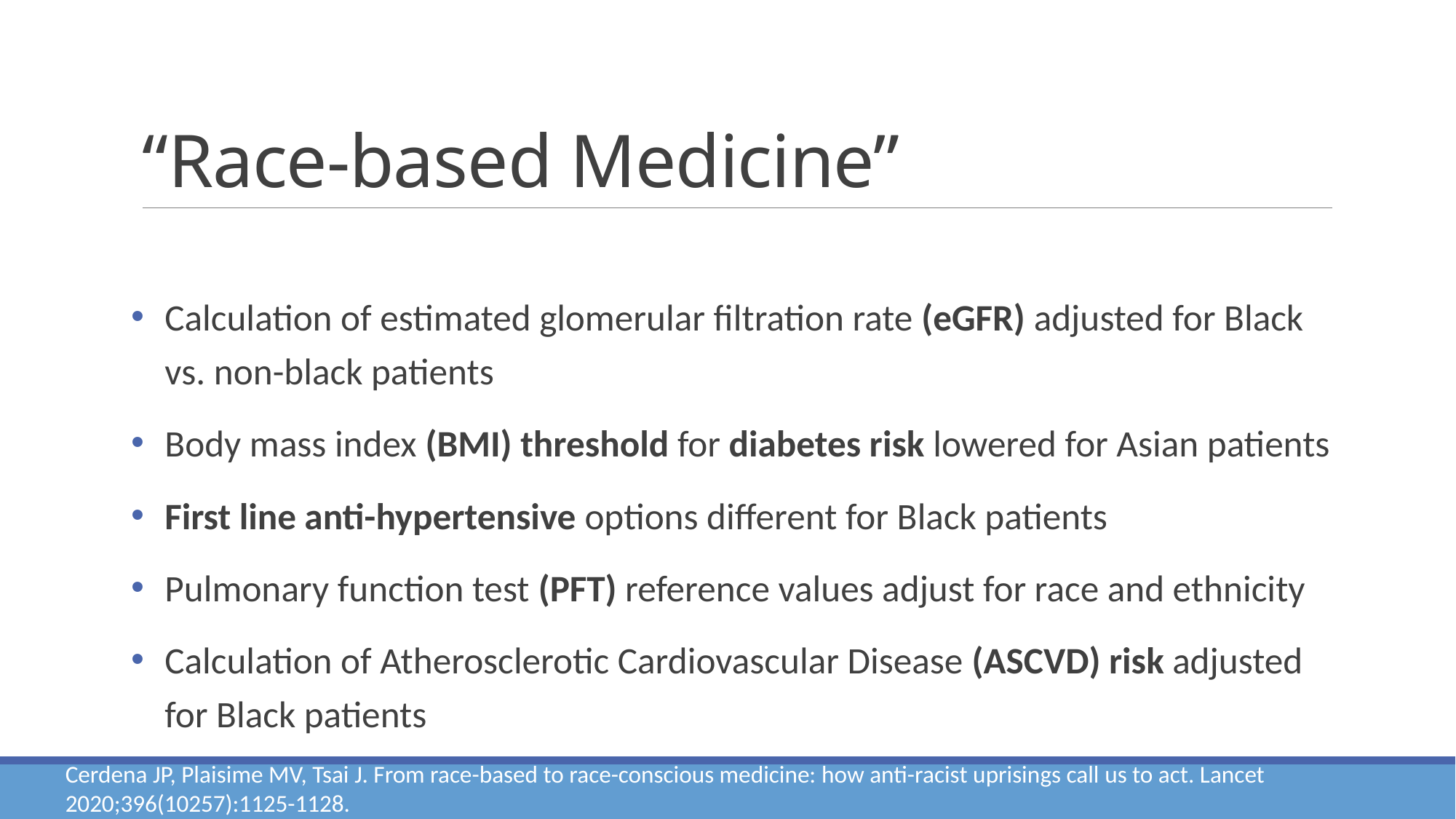

# “Race-based Medicine”
Calculation of estimated glomerular filtration rate (eGFR) adjusted for Black vs. non-black patients
Body mass index (BMI) threshold for diabetes risk lowered for Asian patients
First line anti-hypertensive options different for Black patients
Pulmonary function test (PFT) reference values adjust for race and ethnicity
Calculation of Atherosclerotic Cardiovascular Disease (ASCVD) risk adjusted for Black patients
Cerdena JP, Plaisime MV, Tsai J. From race-based to race-conscious medicine: how anti-racist uprisings call us to act. Lancet 2020;396(10257):1125-1128.

## Slide 8
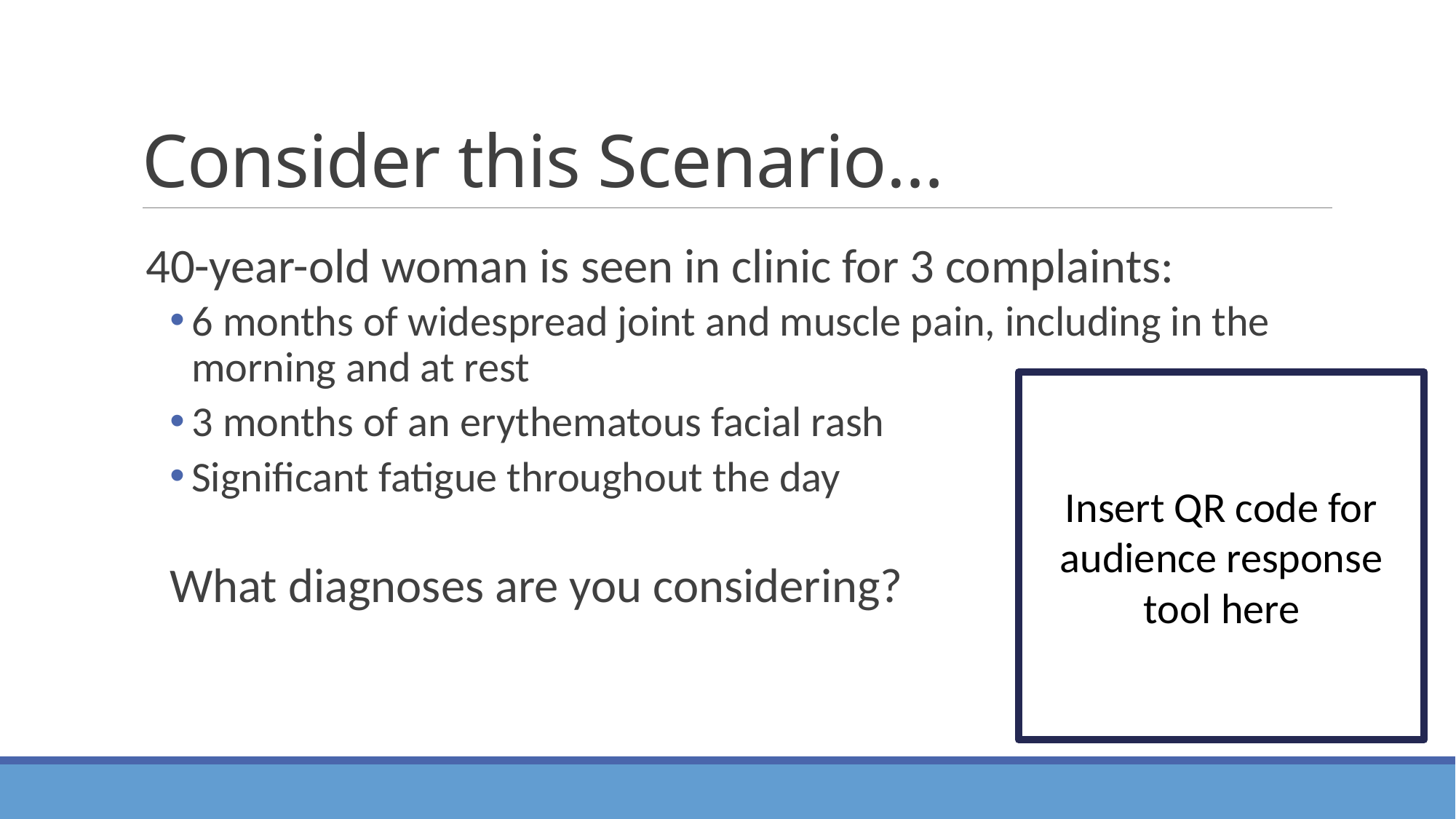

# Consider this Scenario…
40-year-old woman is seen in clinic for 3 complaints:
6 months of widespread joint and muscle pain, including in the morning and at rest
3 months of an erythematous facial rash
Significant fatigue throughout the day
What diagnoses are you considering?
Insert QR code for audience response tool here

## Slide 9
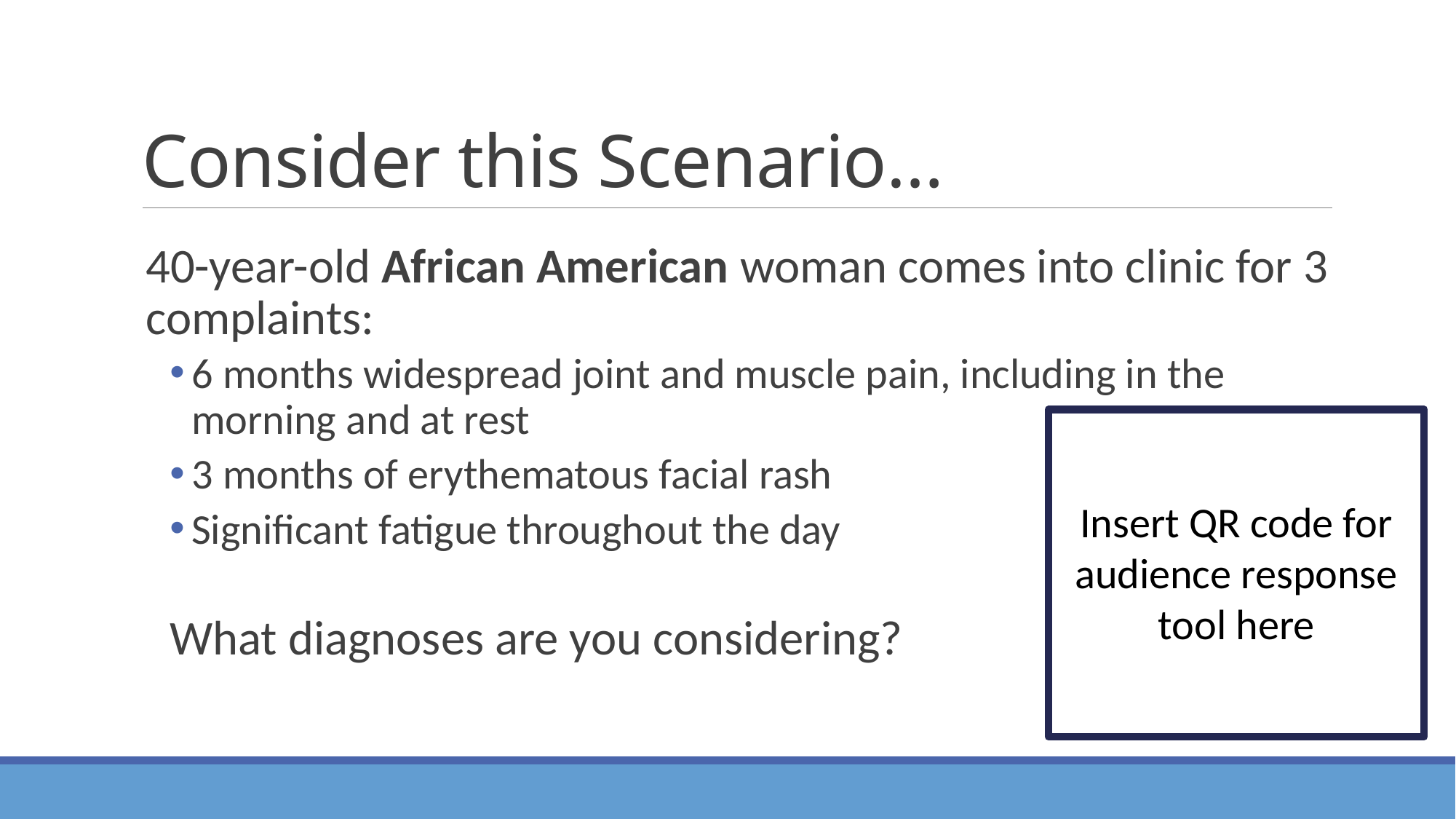

# Consider this Scenario…
40-year-old African American woman comes into clinic for 3 complaints:
6 months widespread joint and muscle pain, including in the morning and at rest
3 months of erythematous facial rash
Significant fatigue throughout the day
What diagnoses are you considering?
Insert QR code for audience response tool here

## Slide 10
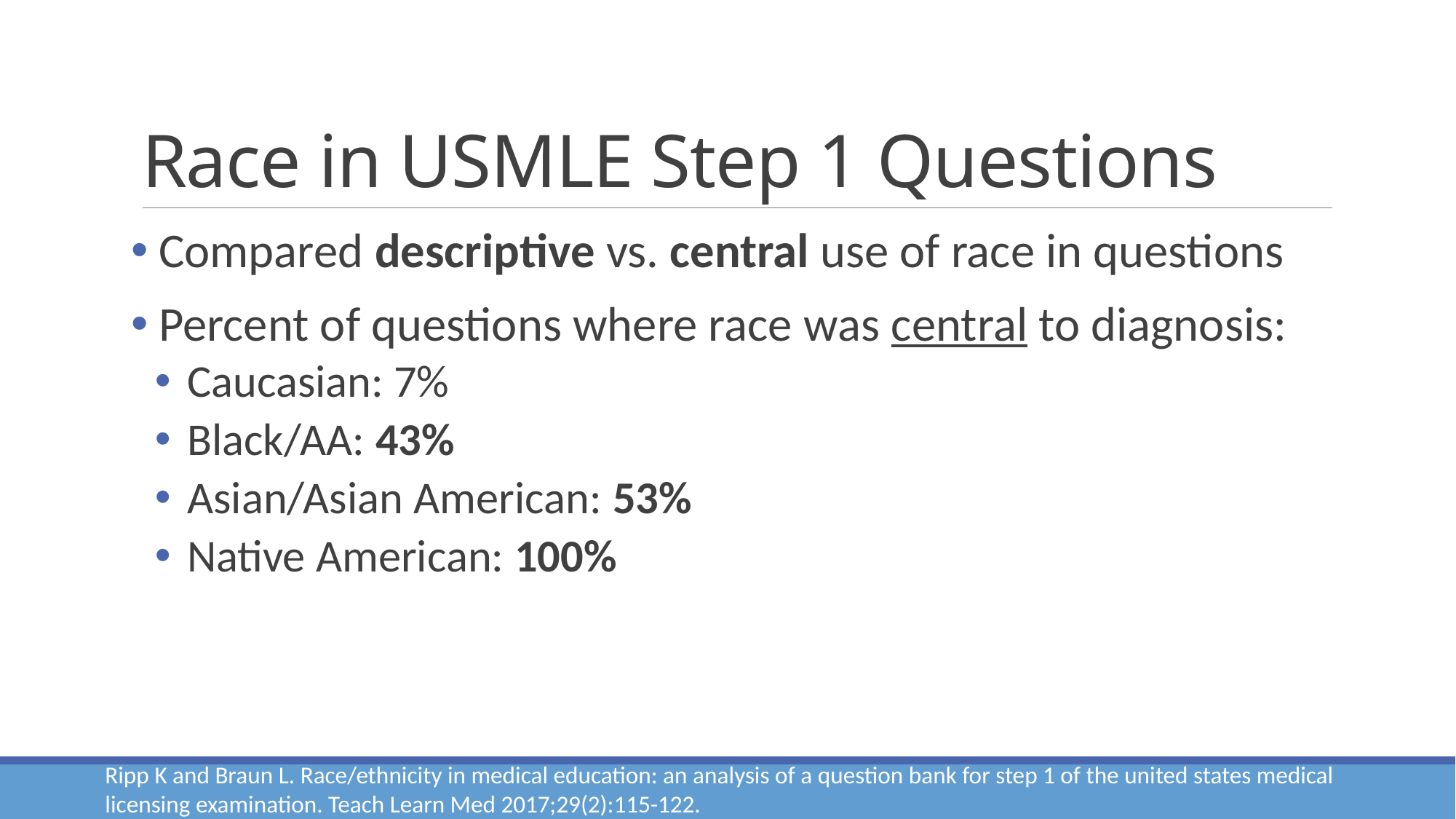

# Race in USMLE Step 1 Questions
 Compared descriptive vs. central use of race in questions
 Percent of questions where race was central to diagnosis:
 Caucasian: 7%
 Black/AA: 43%
 Asian/Asian American: 53%
 Native American: 100%
Ripp K and Braun L. Race/ethnicity in medical education: an analysis of a question bank for step 1 of the united states medical licensing examination. Teach Learn Med 2017;29(2):115-122.

## Slide 11
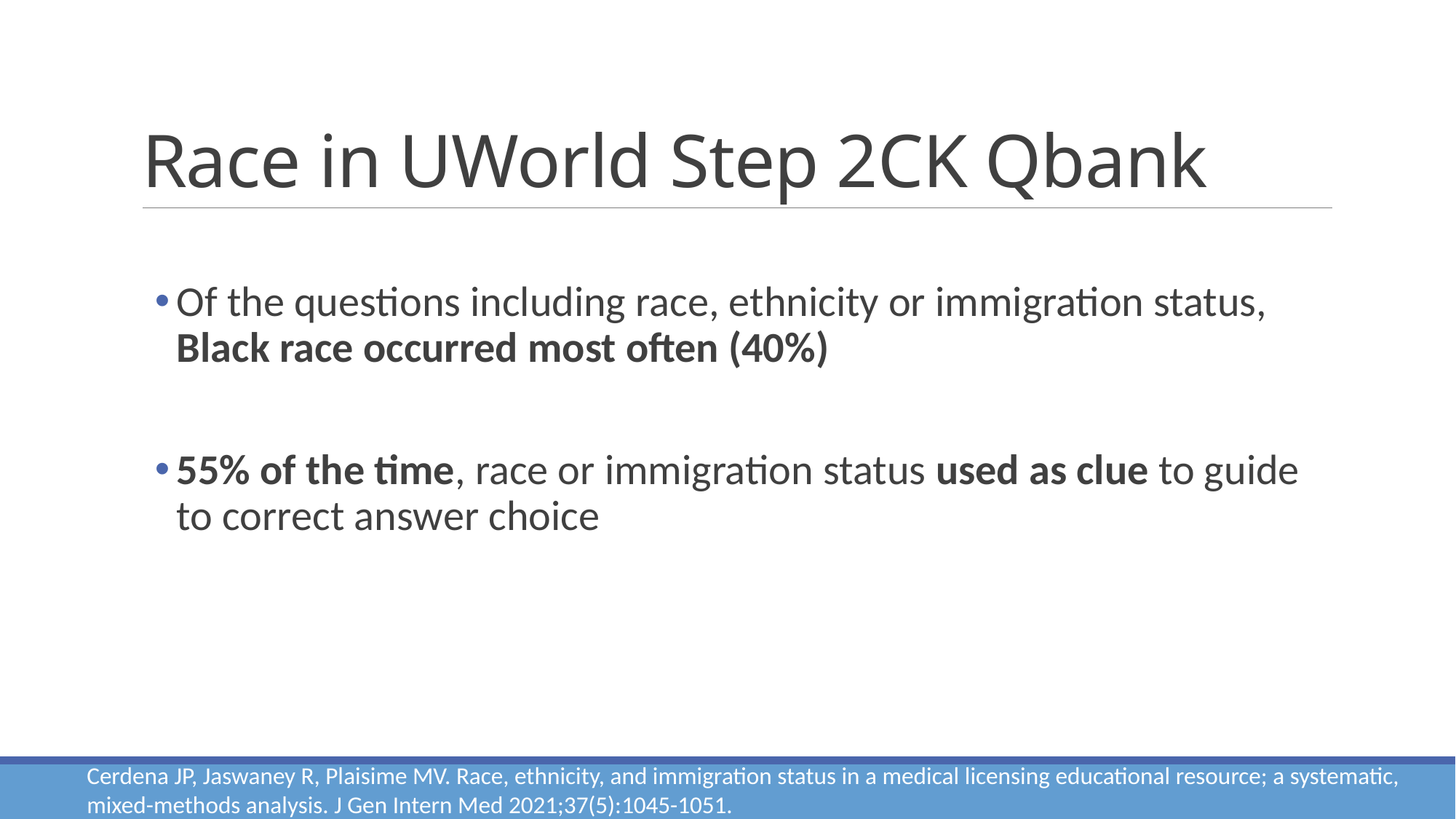

# Race in UWorld Step 2CK Qbank
Of the questions including race, ethnicity or immigration status, Black race occurred most often (40%)
55% of the time, race or immigration status used as clue to guide to correct answer choice
Cerdena JP, Jaswaney R, Plaisime MV. Race, ethnicity, and immigration status in a medical licensing educational resource; a systematic, mixed-methods analysis. J Gen Intern Med 2021;37(5):1045-1051.

## Slide 12
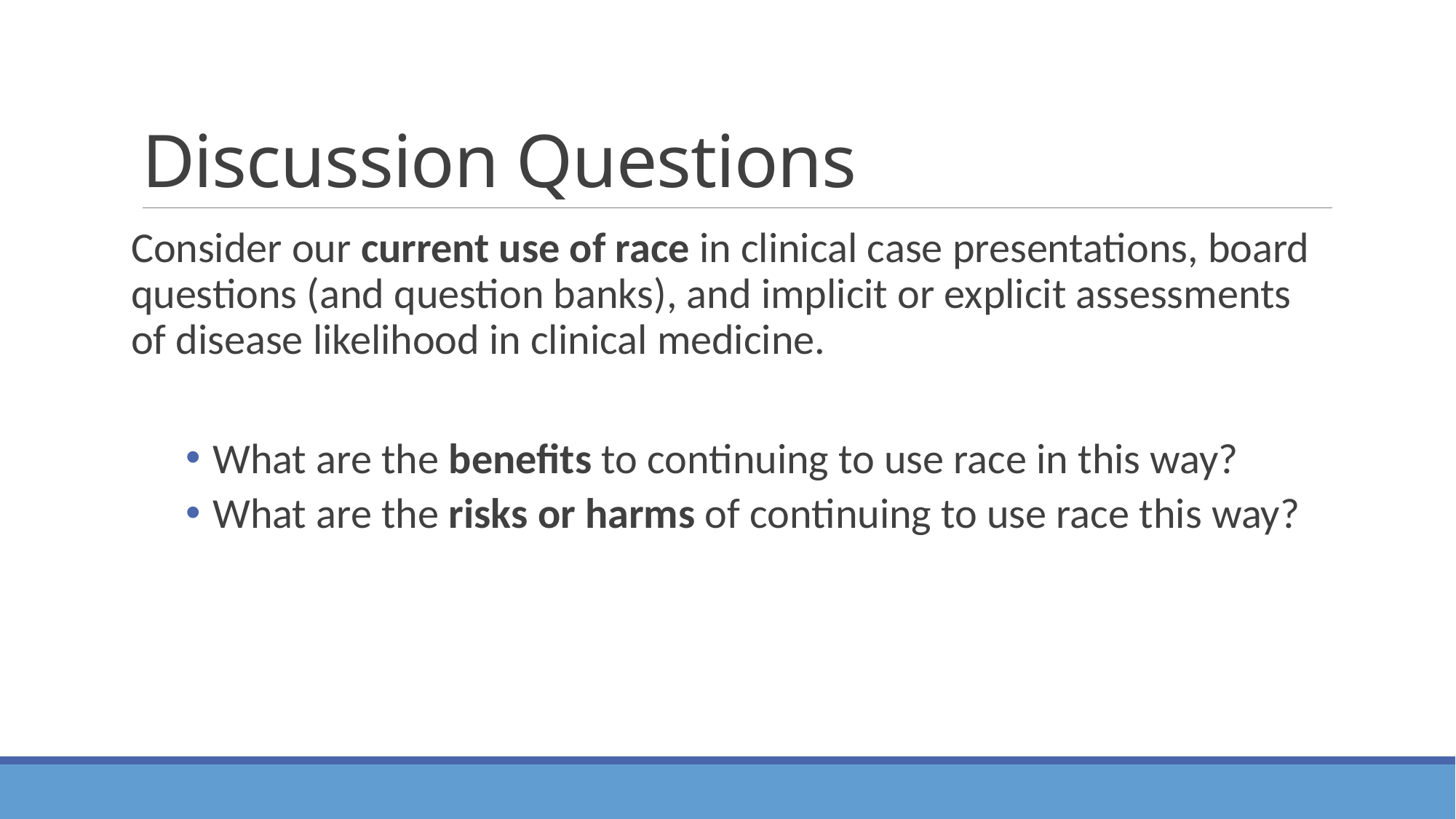

# Discussion Questions
Consider our current use of race in clinical case presentations, board questions (and question banks), and implicit or explicit assessments of disease likelihood in clinical medicine.
What are the benefits to continuing to use race in this way?
What are the risks or harms of continuing to use race this way?

## Slide 13
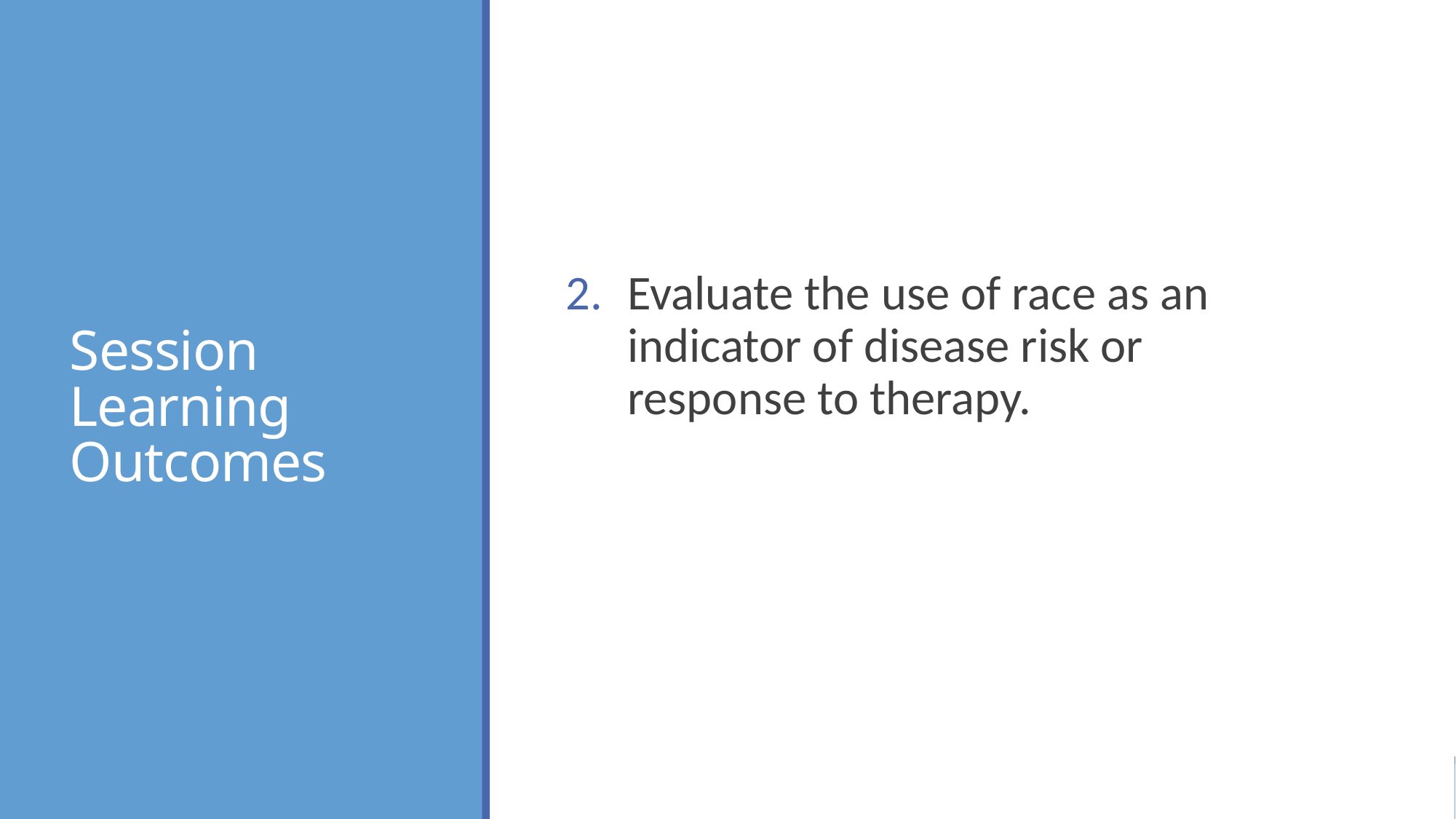

# Session Learning Outcomes
Recognize the ways race is used as a proxy for genetic determinants of health.
Evaluate the use of race as an indicator of disease risk or response to therapy.
Assess the risks and benefits of the ongoing practice of race-based medicine.
Discuss alternate ways of considering race in medicine.

## Slide 14
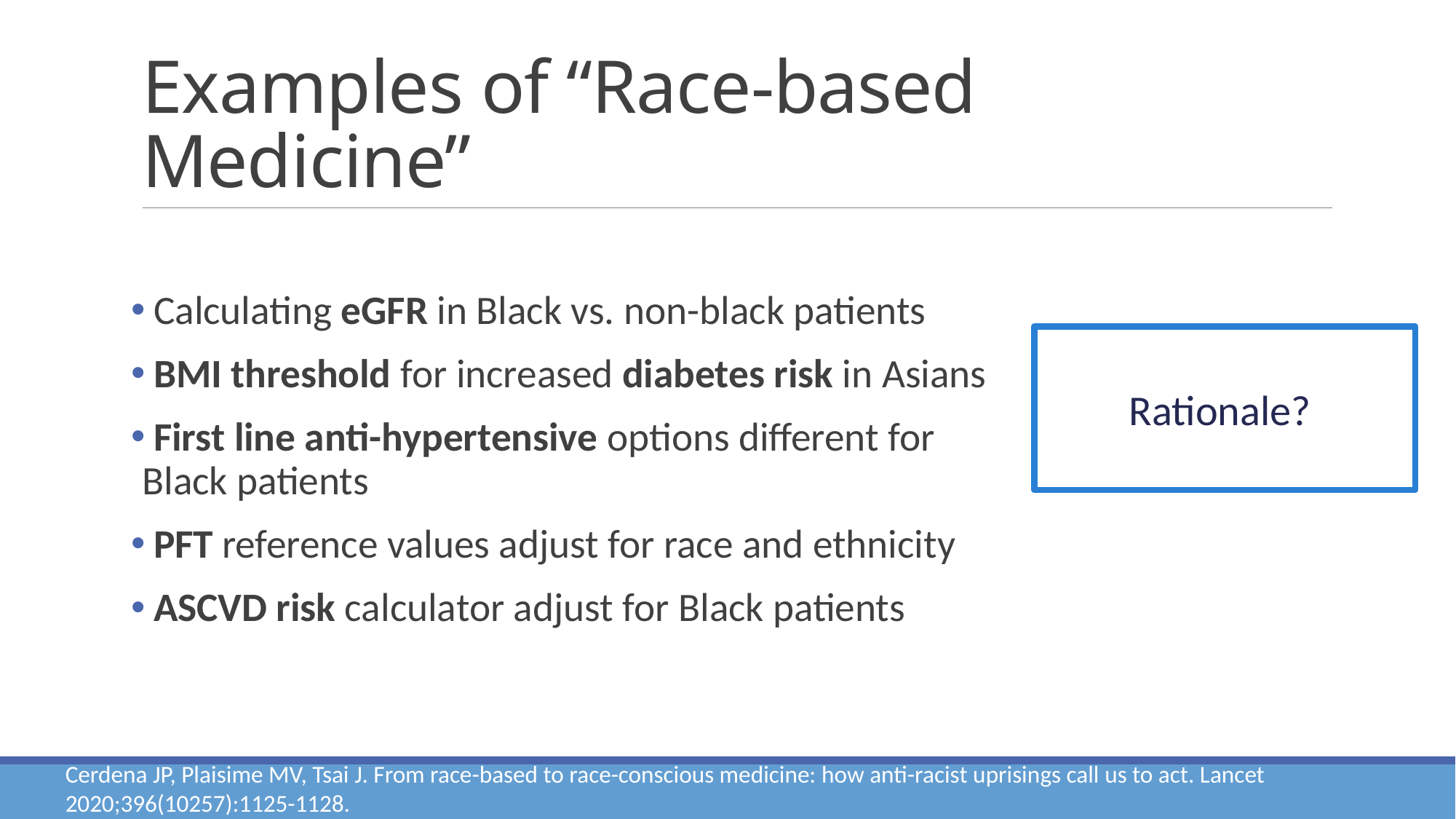

# Examples of “Race-based Medicine”
 Calculating eGFR in Black vs. non-black patients
 BMI threshold for increased diabetes risk in Asians
 First line anti-hypertensive options different for Black patients
 PFT reference values adjust for race and ethnicity
 ASCVD risk calculator adjust for Black patients
Rationale?
Cerdena JP, Plaisime MV, Tsai J. From race-based to race-conscious medicine: how anti-racist uprisings call us to act. Lancet 2020;396(10257):1125-1128.

## Slide 15
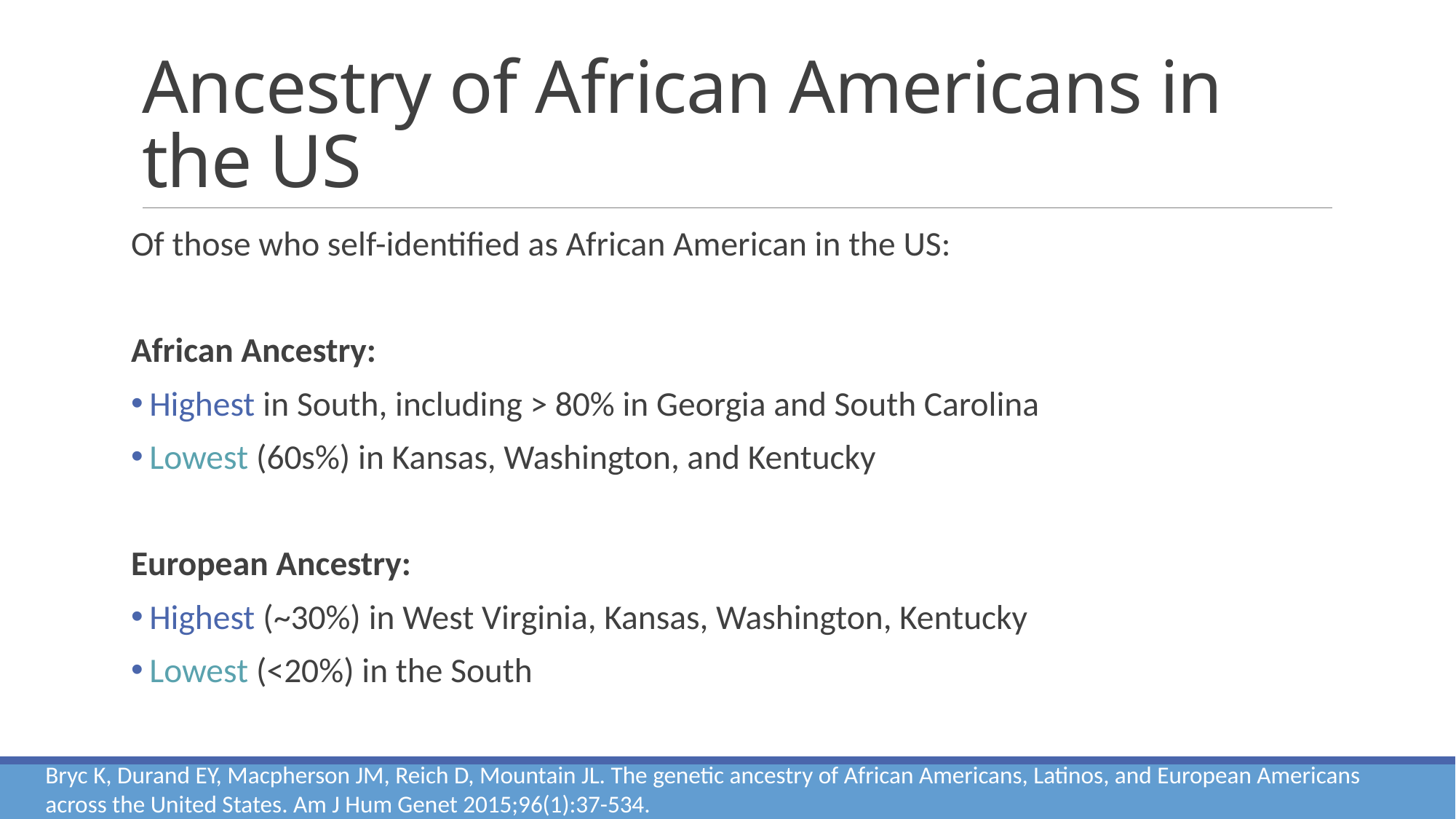

# Ancestry of African Americans in the US
Of those who self-identified as African American in the US:
African Ancestry:
 Highest in South, including > 80% in Georgia and South Carolina
 Lowest (60s%) in Kansas, Washington, and Kentucky
European Ancestry:
 Highest (~30%) in West Virginia, Kansas, Washington, Kentucky
 Lowest (<20%) in the South
Bryc K, Durand EY, Macpherson JM, Reich D, Mountain JL. The genetic ancestry of African Americans, Latinos, and European Americans across the United States. Am J Hum Genet 2015;96(1):37-534.

## Slide 16
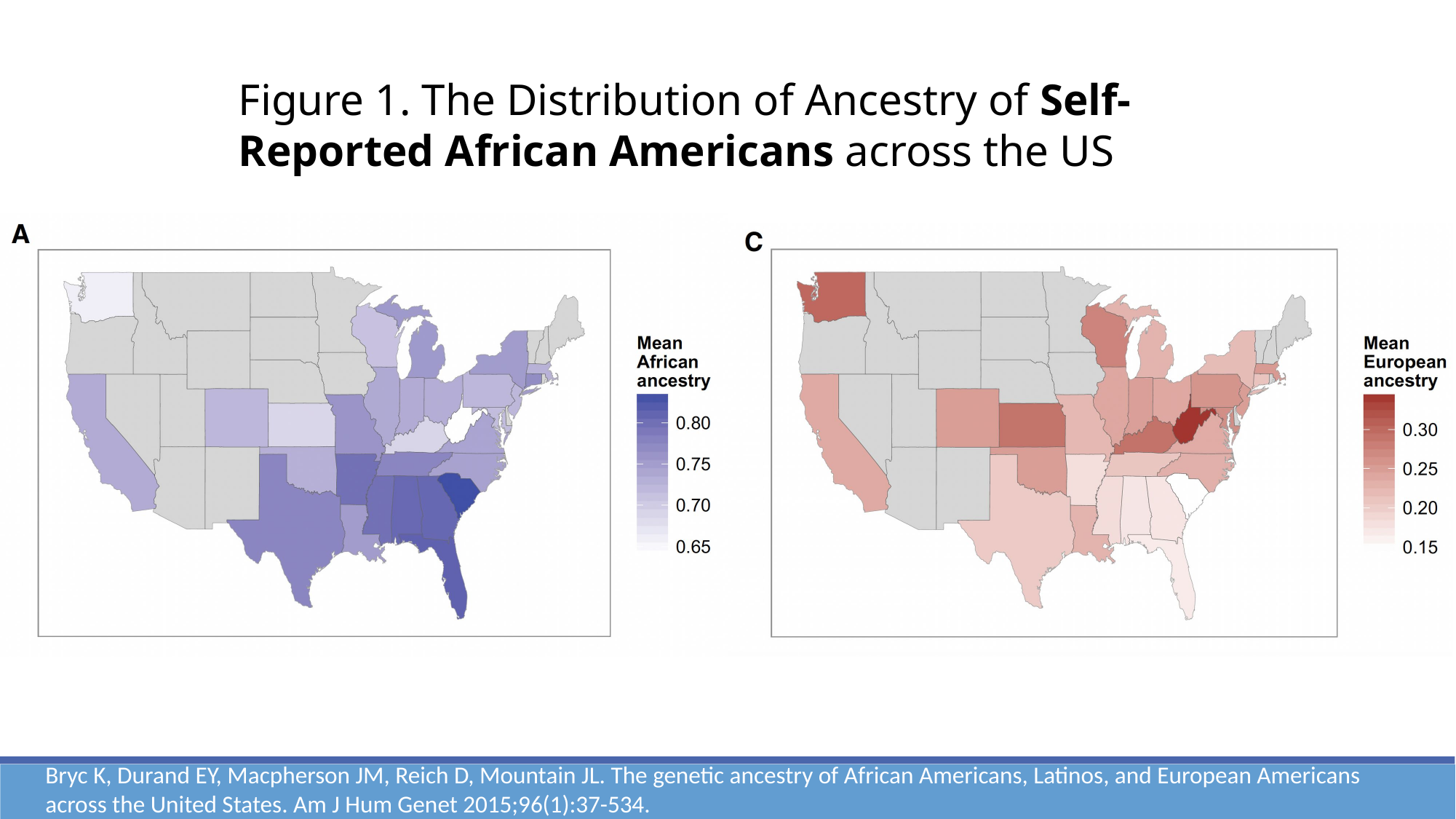

Figure 1. The Distribution of Ancestry of Self-Reported African Americans across the US
Bryc K, Durand EY, Macpherson JM, Reich D, Mountain JL. The genetic ancestry of African Americans, Latinos, and European Americans across the United States. Am J Hum Genet 2015;96(1):37-534.

## Slide 17
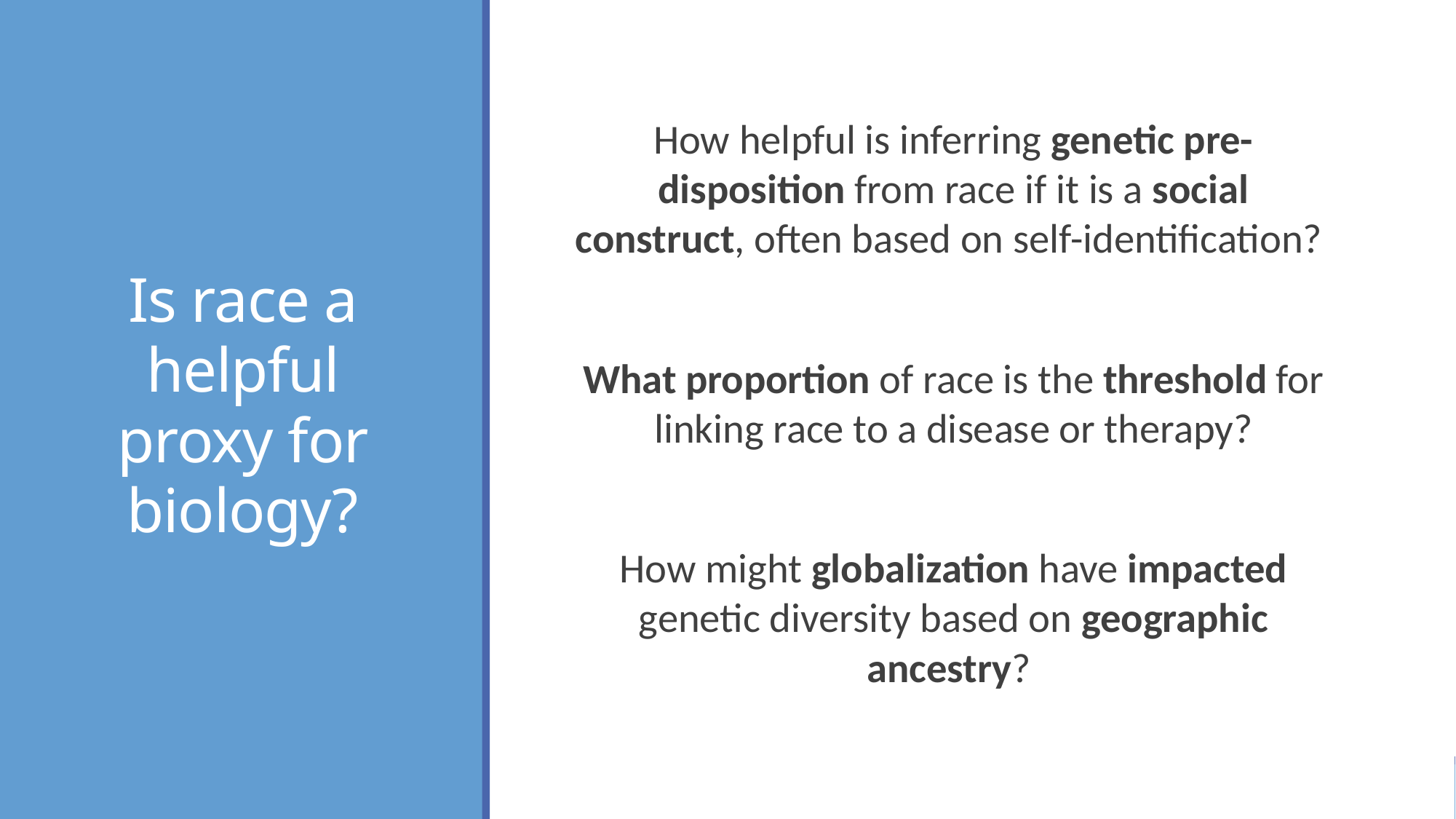

# Is race a helpful proxy for biology?
How helpful is inferring genetic pre-disposition from race if it is a social construct, often based on self-identification?
What proportion of race is the threshold for linking race to a disease or therapy?
How might globalization have impacted genetic diversity based on geographic ancestry?

## Slide 18
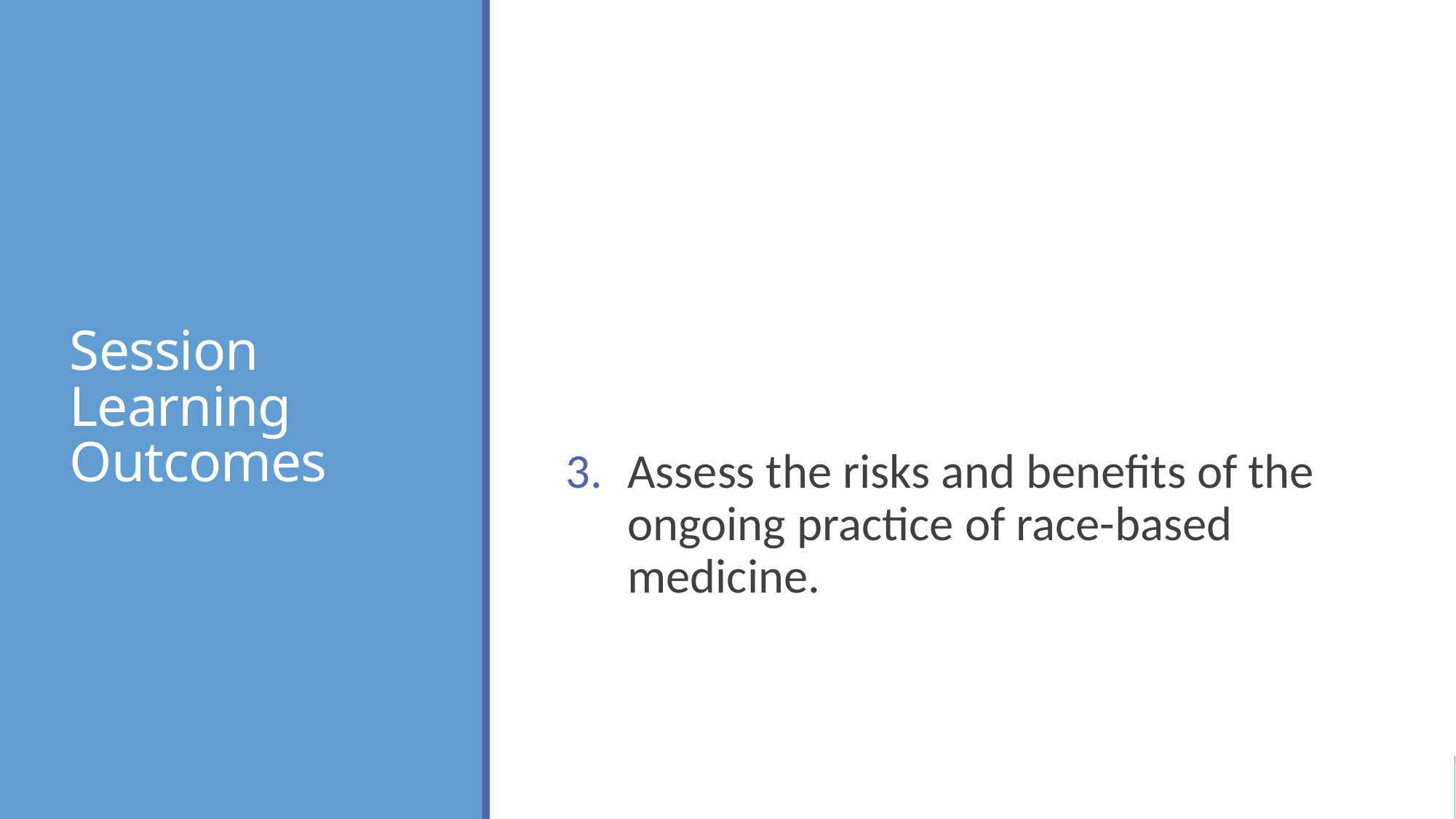

# Session Learning Outcomes
Recognize the ways race is used as a proxy for genetic determinants of health.
Evaluate the use of race as an indicator of disease risk or response to therapy.
Assess the risks and benefits of the ongoing practice of race-based medicine.
Describe evolving frameworks for considering race in medicine.

## Slide 19
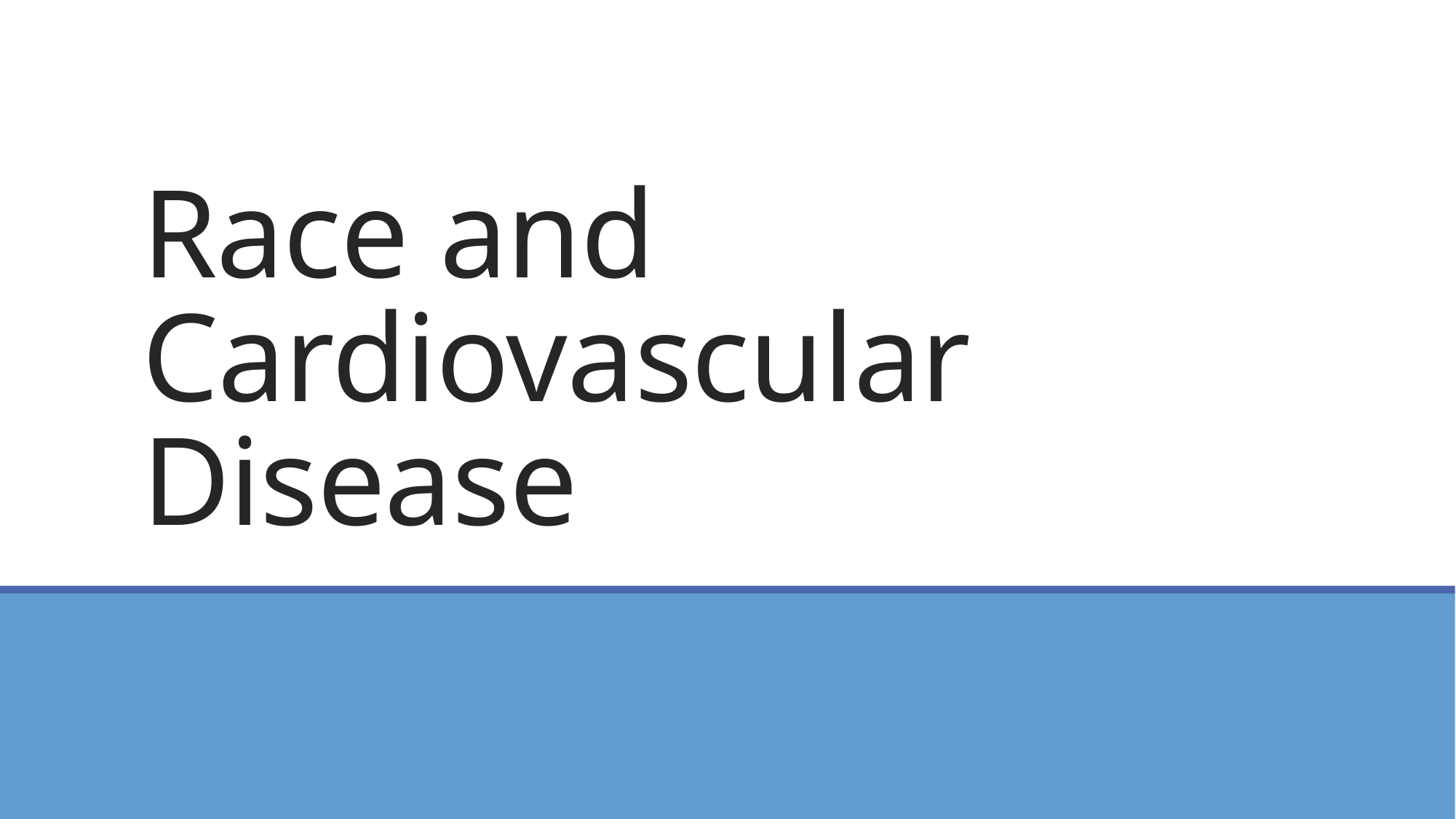

# Race and Cardiovascular Disease

## Slide 20
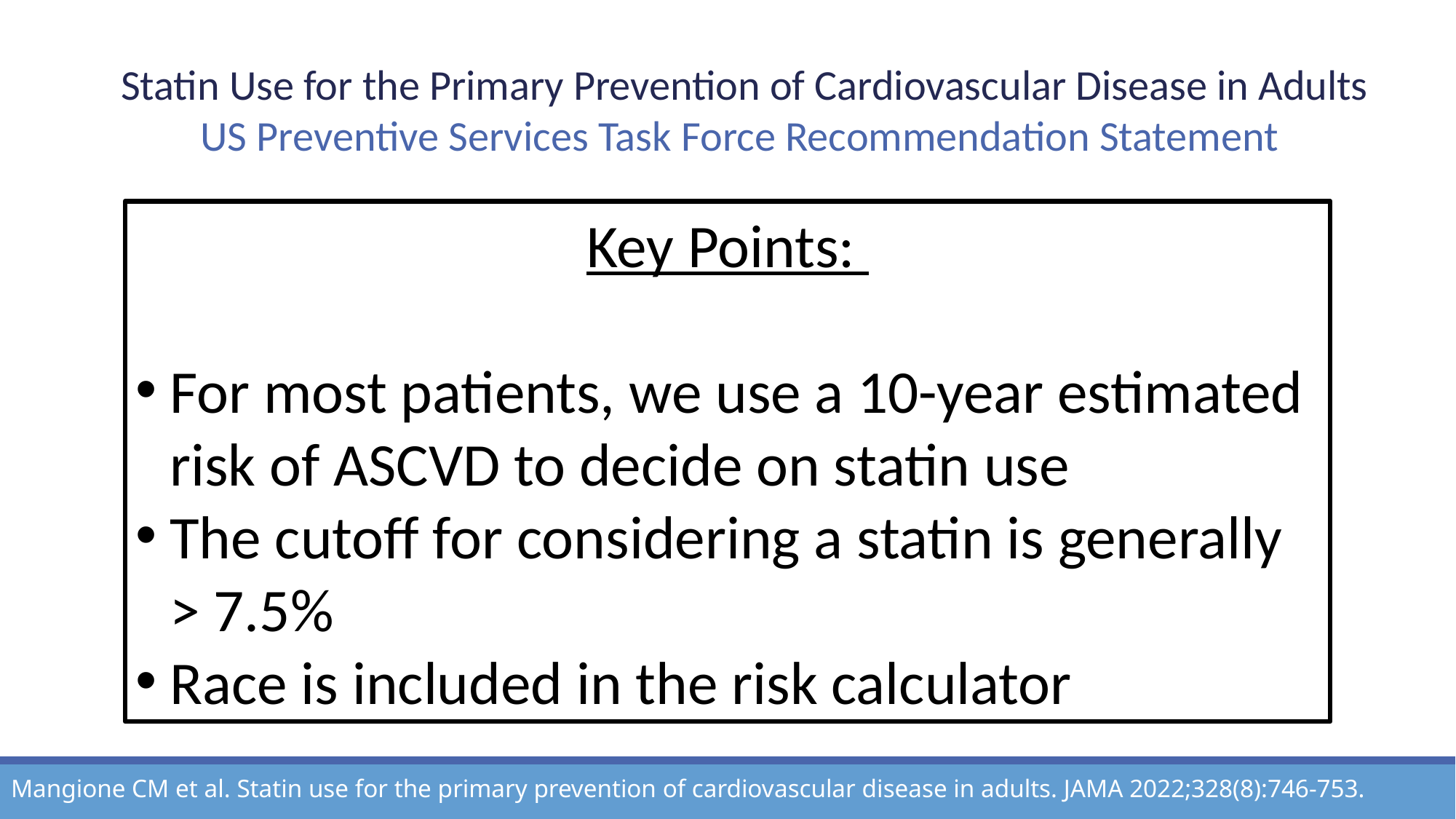

Statin Use for the Primary Prevention of Cardiovascular Disease in Adults
US Preventive Services Task Force Recommendation Statement
Key Points:
For most patients, we use a 10-year estimated risk of ASCVD to decide on statin use
The cutoff for considering a statin is generally > 7.5%
Race is included in the risk calculator
Mangione CM et al. Statin use for the primary prevention of cardiovascular disease in adults. JAMA 2022;328(8):746-753.

## Slide 21
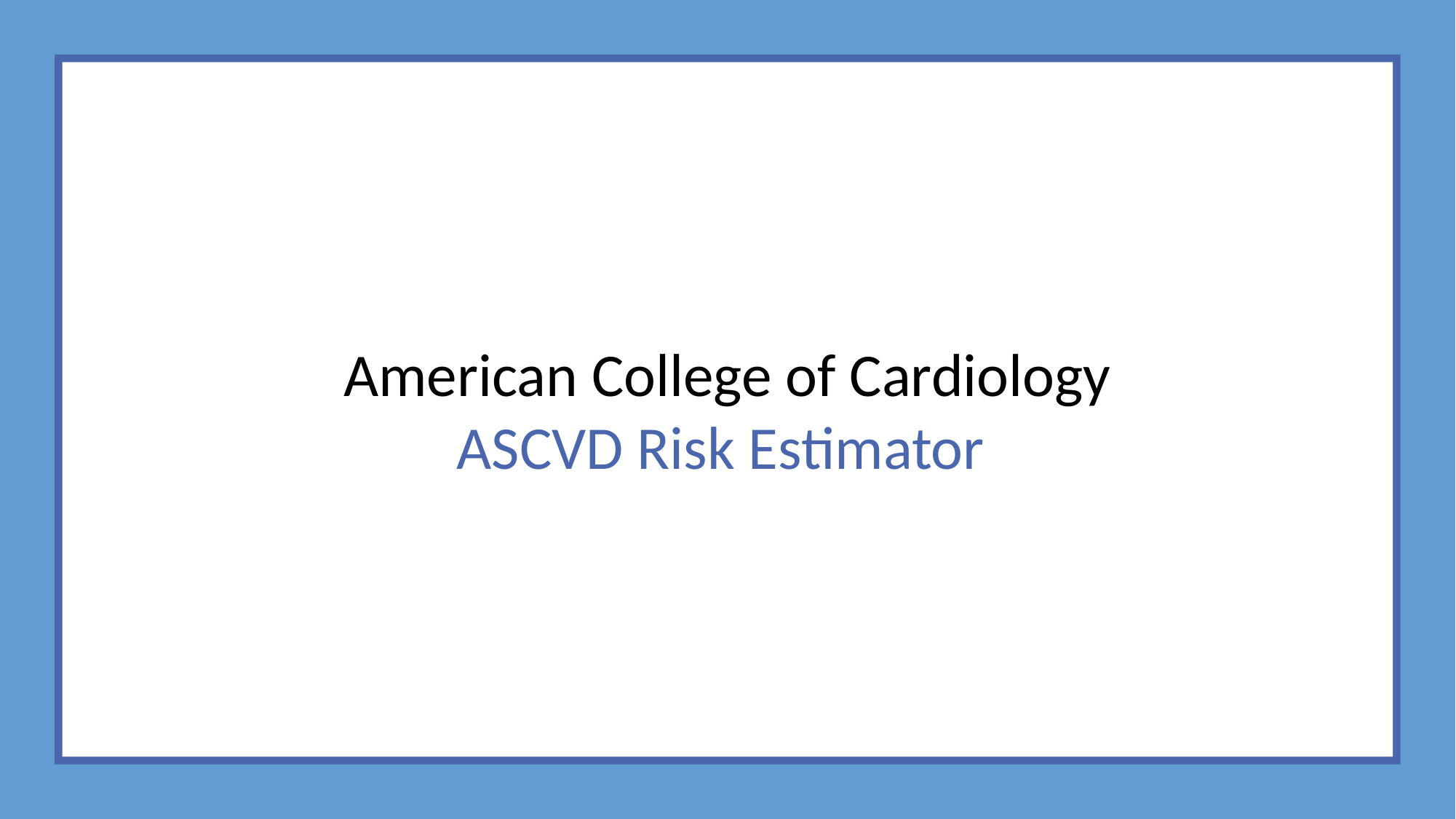

American College of Cardiology
ASCVD Risk Estimator

## Slide 22
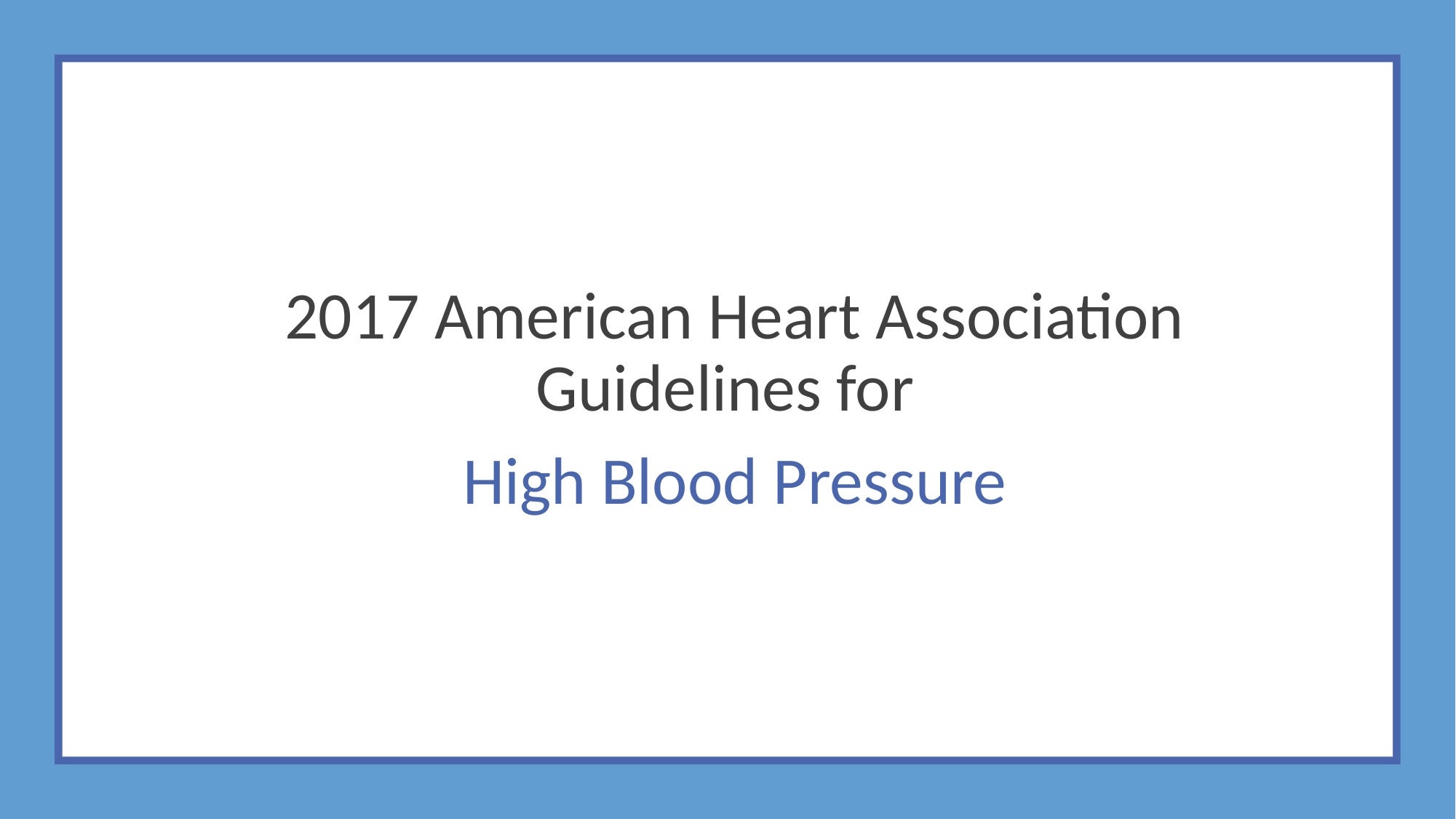

2017 American Heart Association Guidelines for
High Blood Pressure

## Slide 23
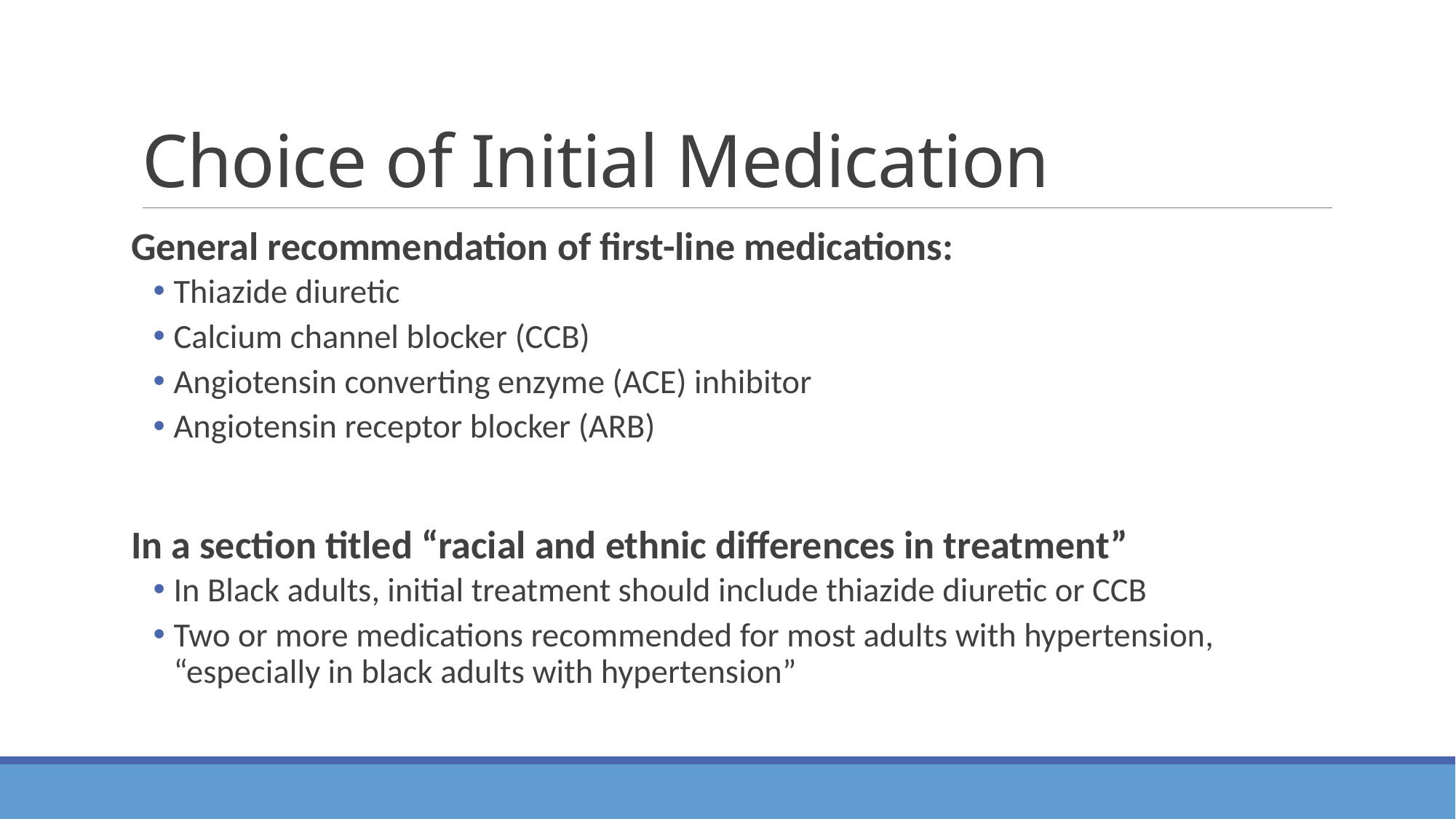

# Choice of Initial Medication
General recommendation of first-line medications:
Thiazide diuretic
Calcium channel blocker (CCB)
Angiotensin converting enzyme (ACE) inhibitor
Angiotensin receptor blocker (ARB)
In a section titled “racial and ethnic differences in treatment”
In Black adults, initial treatment should include thiazide diuretic or CCB
Two or more medications recommended for most adults with hypertension, “especially in black adults with hypertension”

## Slide 24
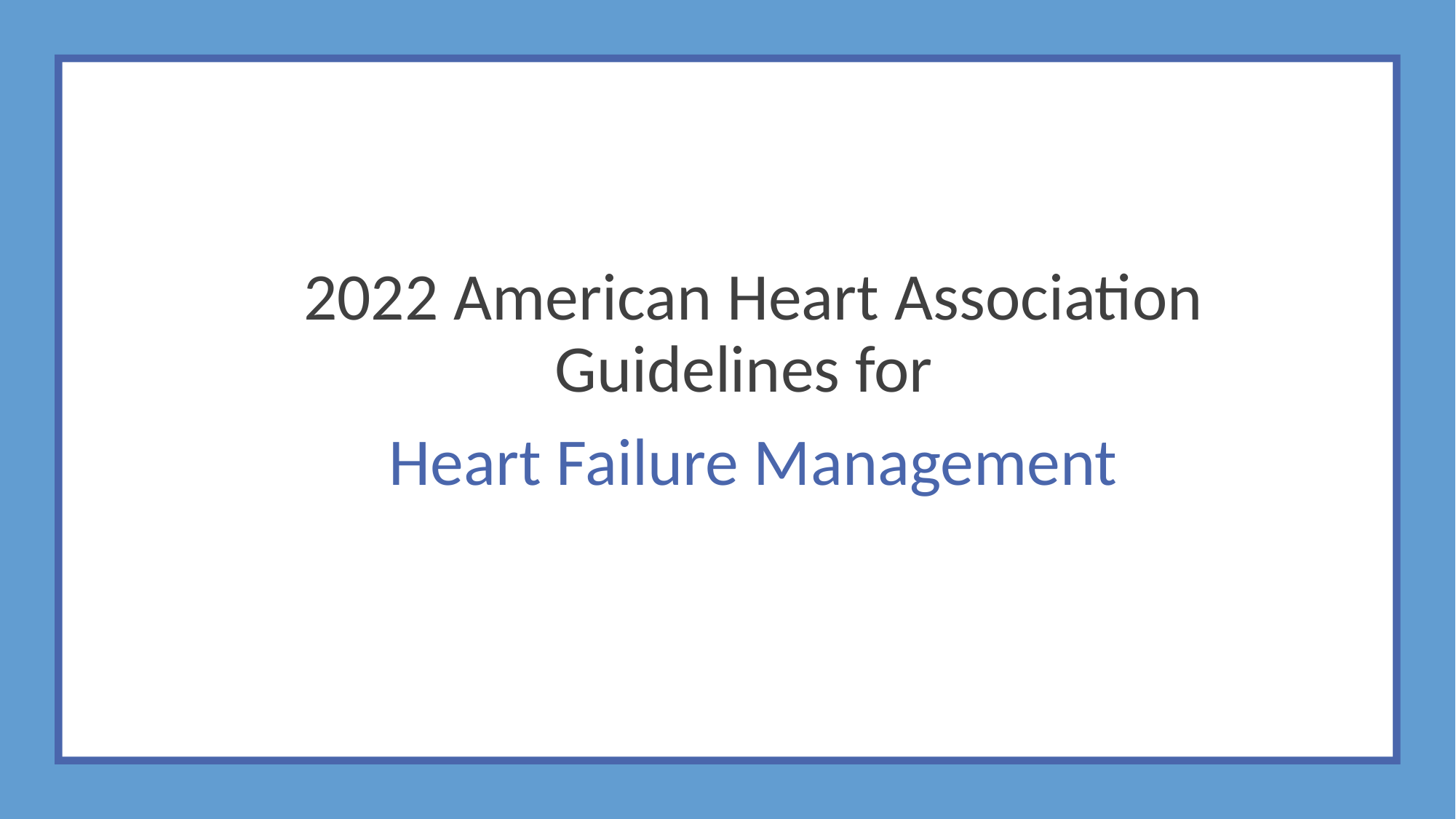

2022 American Heart Association Guidelines for
Heart Failure Management

## Slide 25
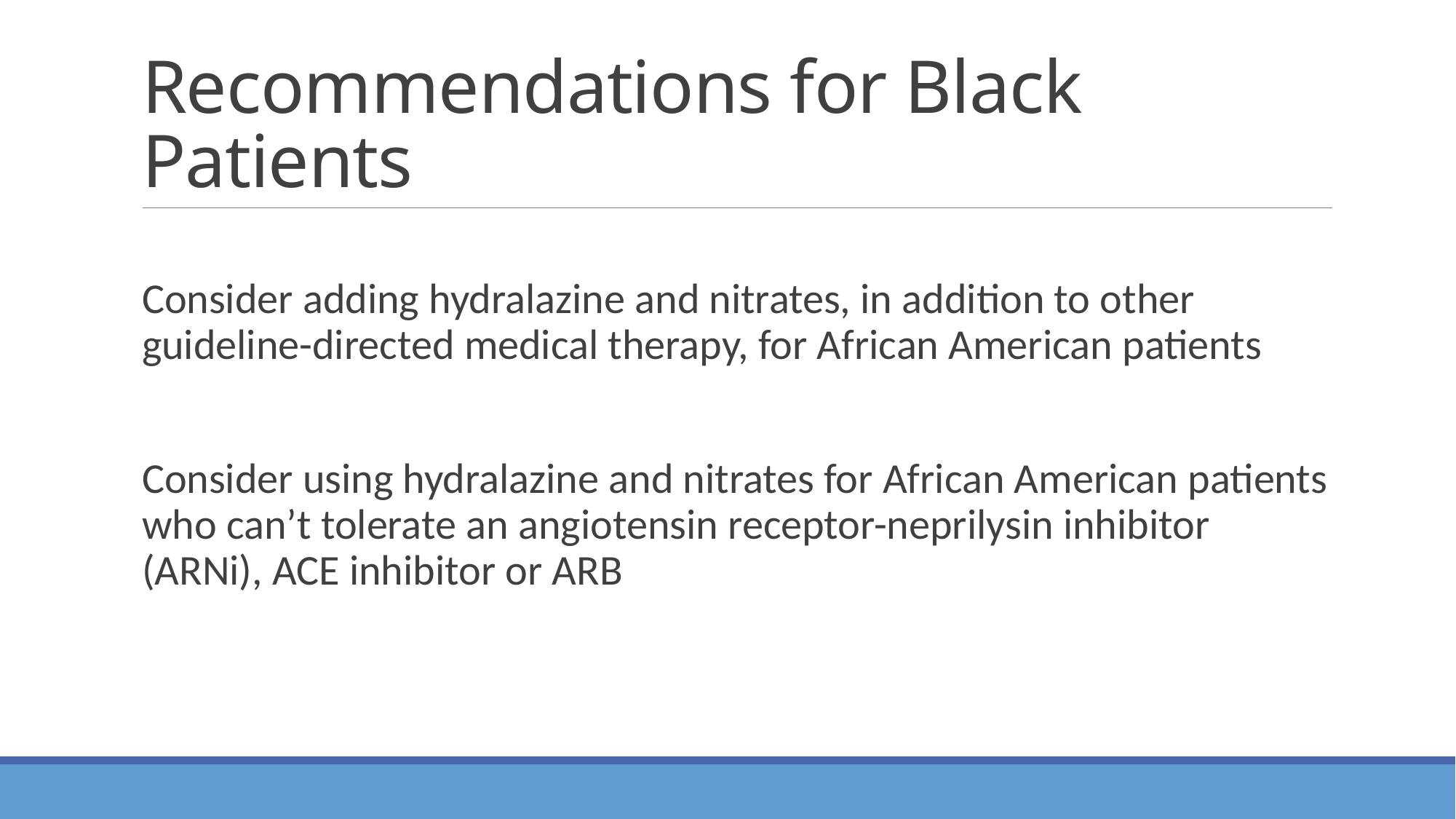

# Recommendations for Black Patients
Consider adding hydralazine and nitrates, in addition to other guideline-directed medical therapy, for African American patients
Consider using hydralazine and nitrates for African American patients who can’t tolerate an angiotensin receptor-neprilysin inhibitor (ARNi), ACE inhibitor or ARB

## Slide 26
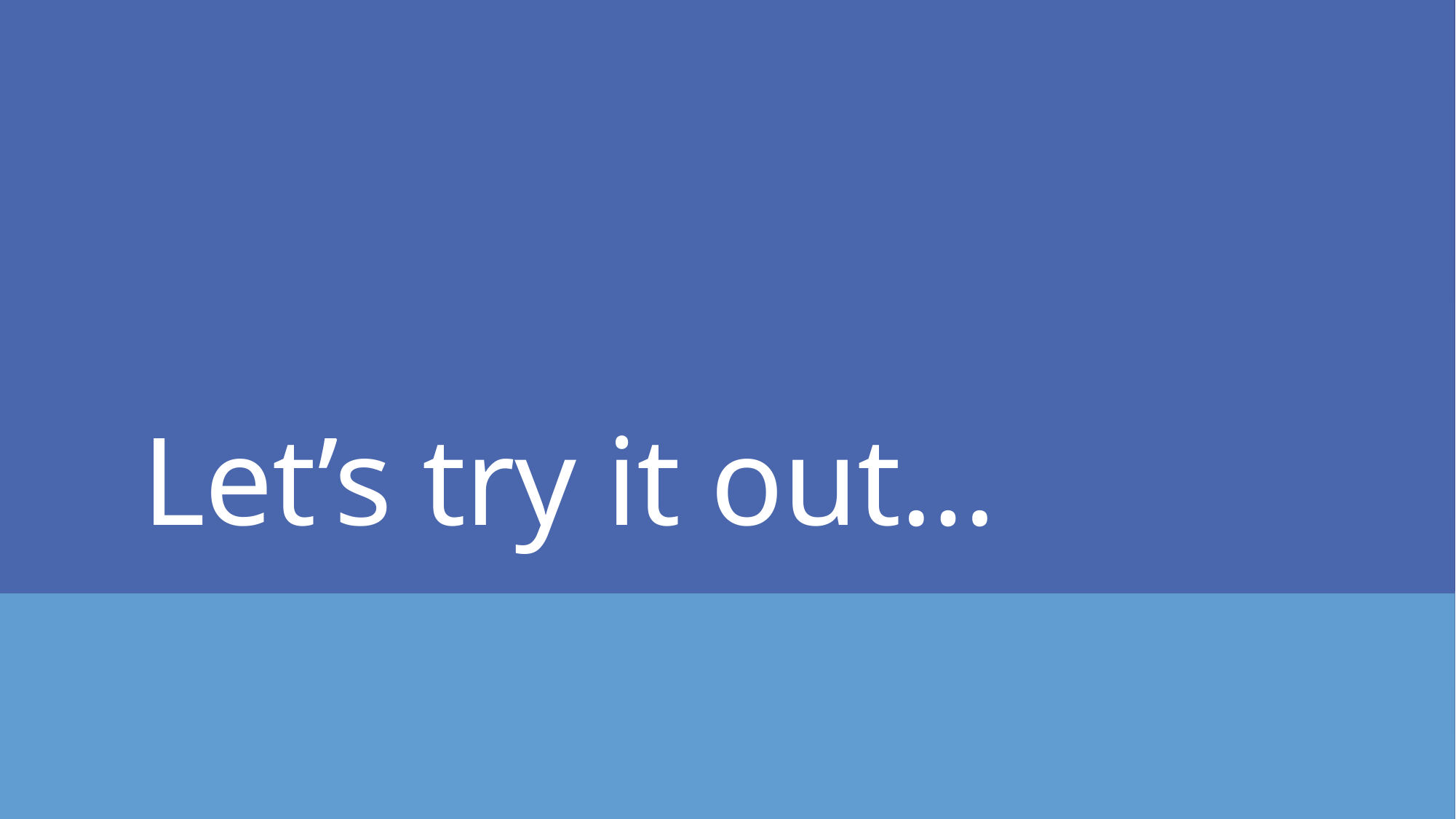

# Let’s try it out…

## Slide 27
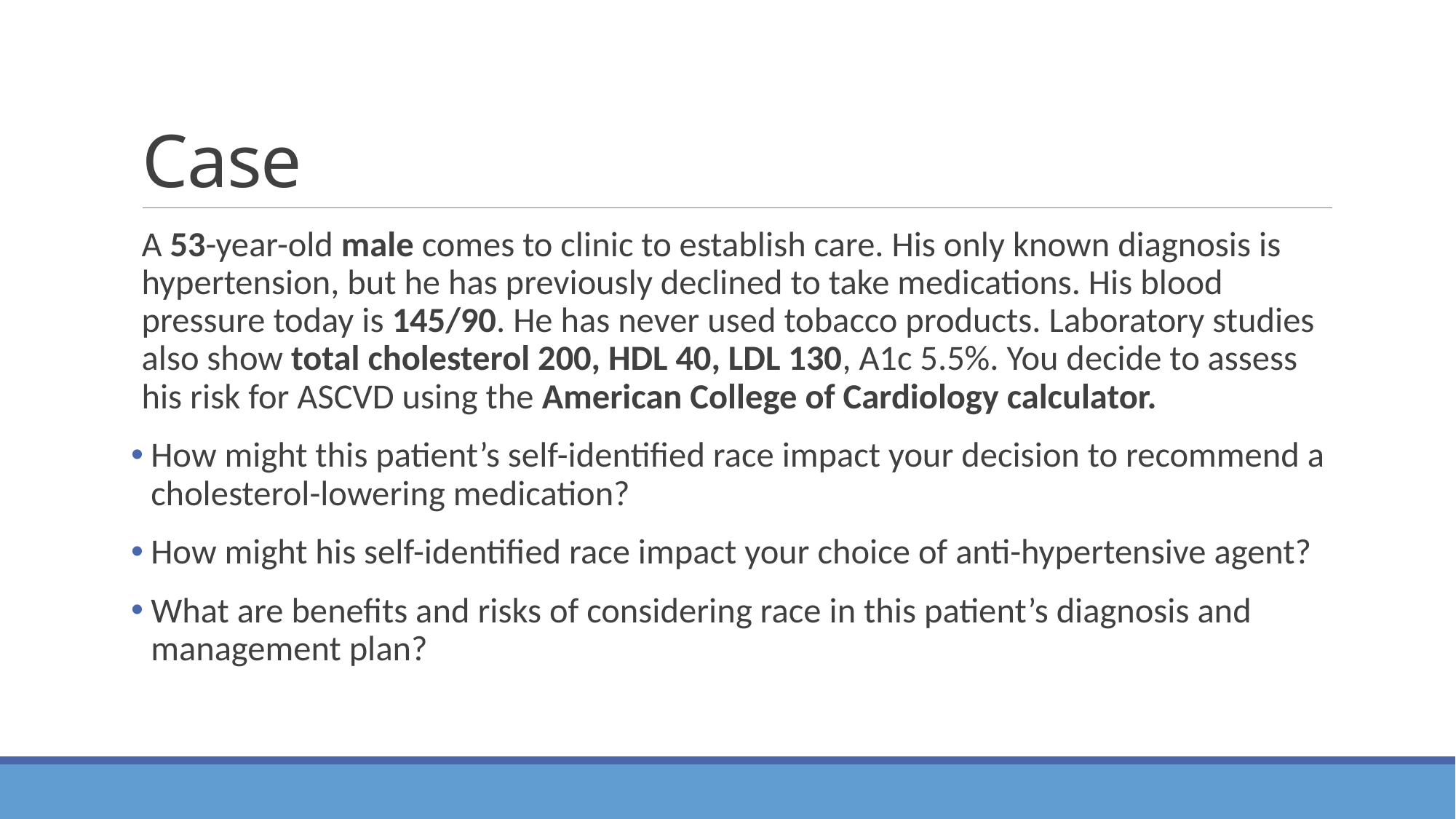

# Case
A 53-year-old male comes to clinic to establish care. His only known diagnosis is hypertension, but he has previously declined to take medications. His blood pressure today is 145/90. He has never used tobacco products. Laboratory studies also show total cholesterol 200, HDL 40, LDL 130, A1c 5.5%. You decide to assess his risk for ASCVD using the American College of Cardiology calculator.
How might this patient’s self-identified race impact your decision to recommend a cholesterol-lowering medication?
How might his self-identified race impact your choice of anti-hypertensive agent?
What are benefits and risks of considering race in this patient’s diagnosis and management plan?

## Slide 28
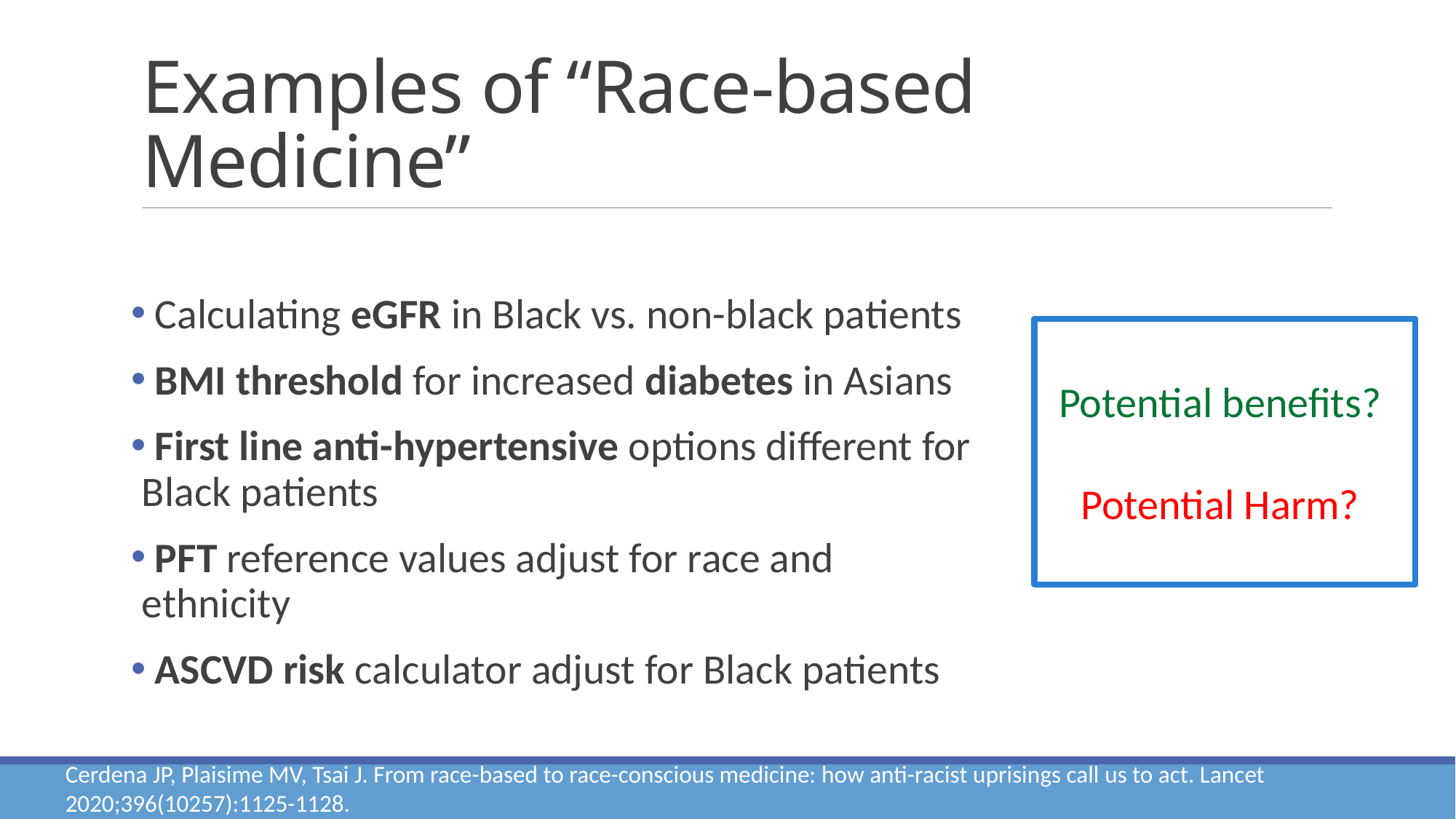

# Examples of “Race-based Medicine”
 Calculating eGFR in Black vs. non-black patients
 BMI threshold for increased diabetes in Asians
 First line anti-hypertensive options different for Black patients
 PFT reference values adjust for race and ethnicity
 ASCVD risk calculator adjust for Black patients
Potential benefits?
Potential Harm?
Cerdena JP, Plaisime MV, Tsai J. From race-based to race-conscious medicine: how anti-racist uprisings call us to act. Lancet 2020;396(10257):1125-1128.

## Slide 29
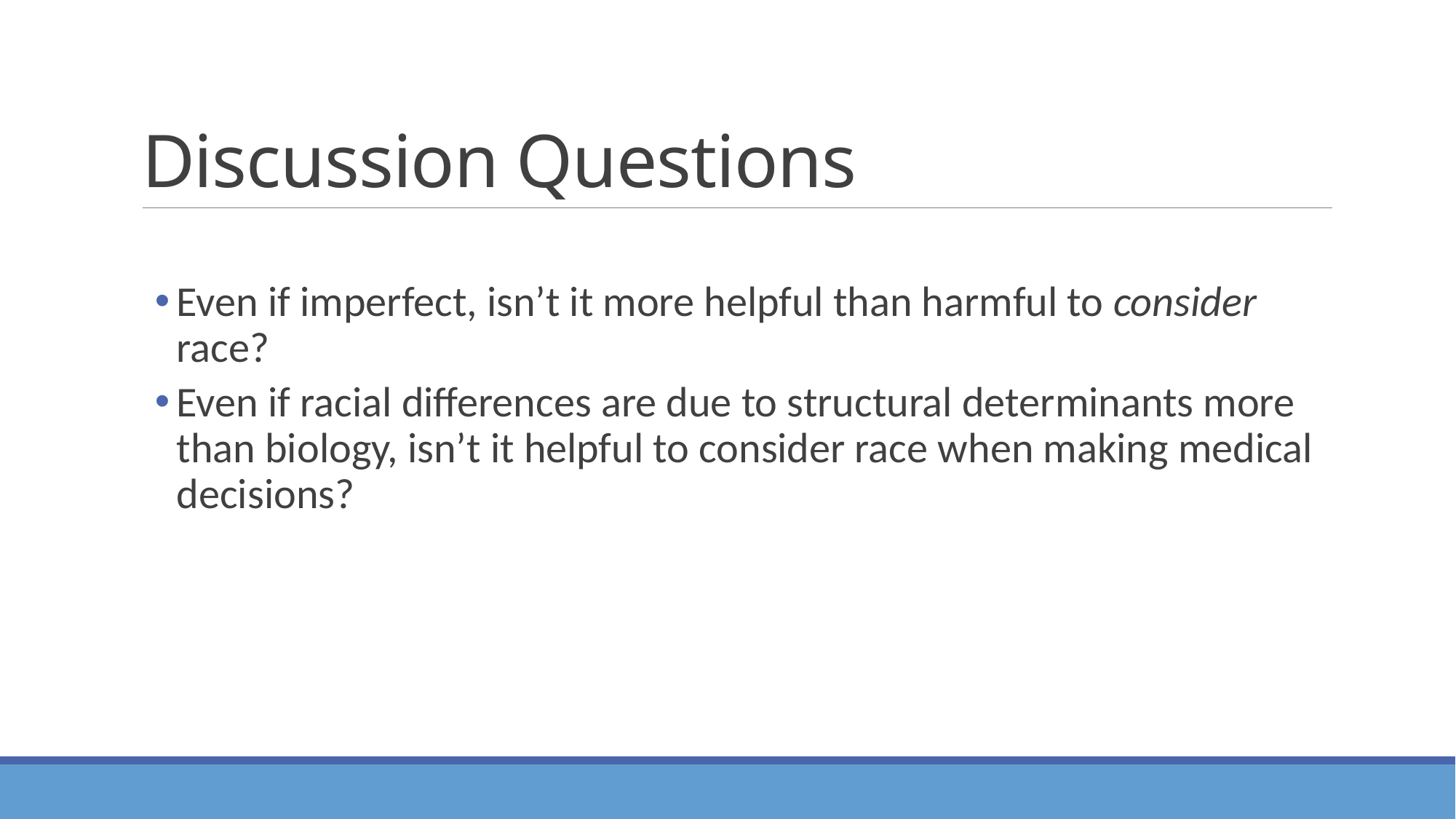

# Discussion Questions
Even if imperfect, isn’t it more helpful than harmful to consider race?
Even if racial differences are due to structural determinants more than biology, isn’t it helpful to consider race when making medical decisions?

## Slide 30
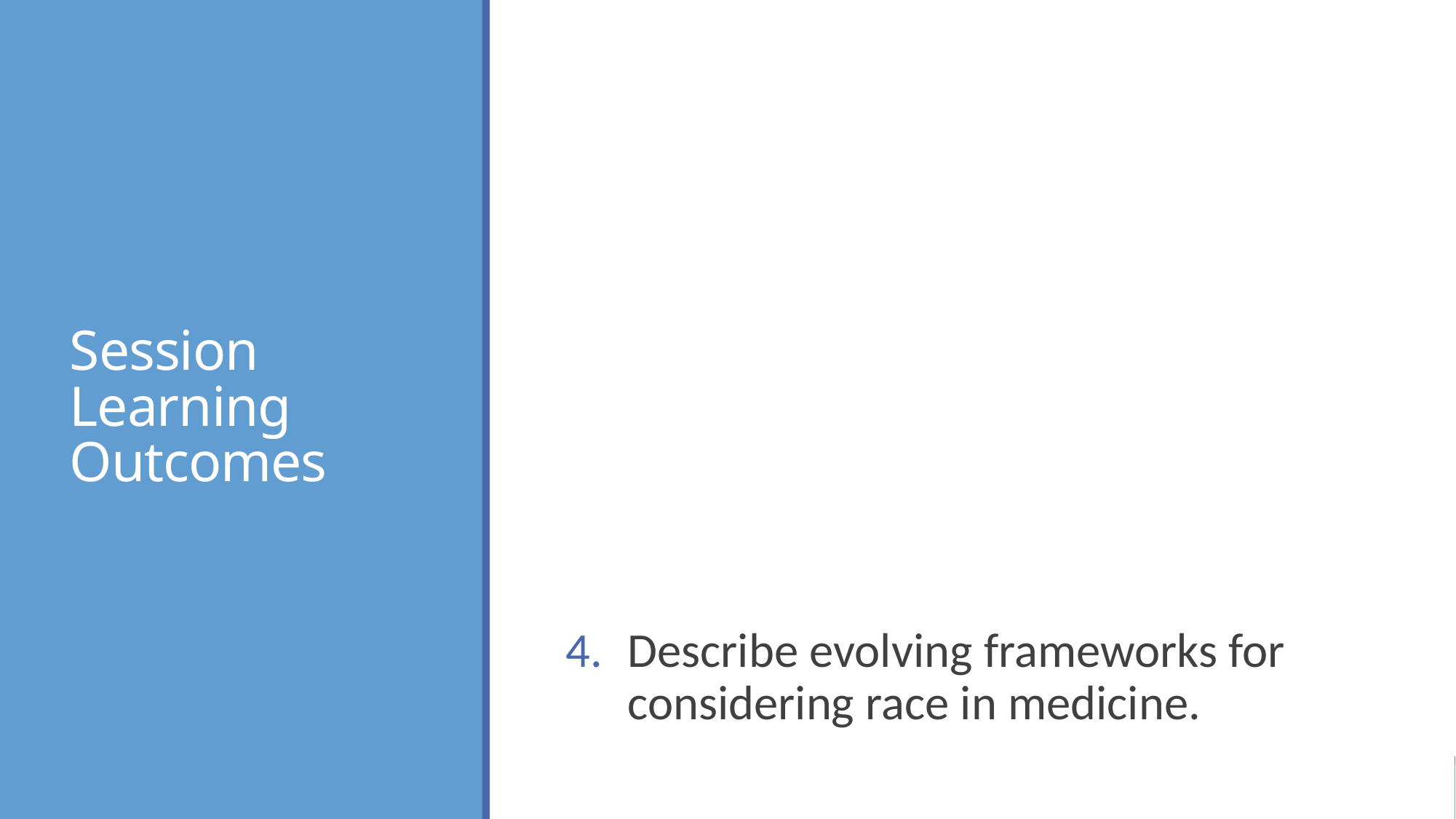

# Session Learning Outcomes
Recognize the ways race is used as a proxy for genetic determinants of health.
Evaluate the use of race as an indicator of disease risk or response to therapy.
Assess the risks and benefits of the ongoing practice of race-based medicine.
Describe evolving frameworks for considering race in medicine.

## Slide 31
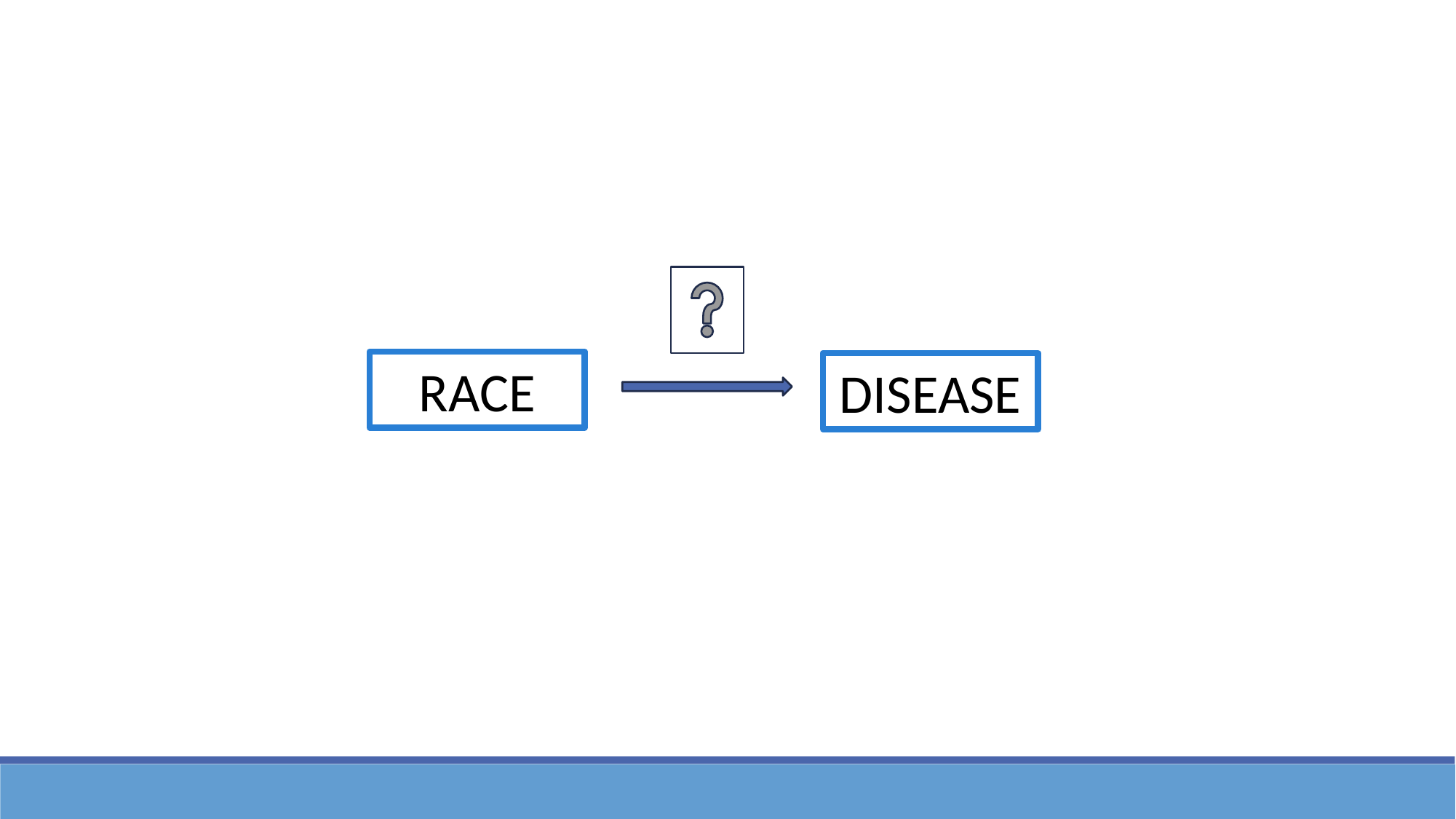

RACE
DISEASE

## Slide 32
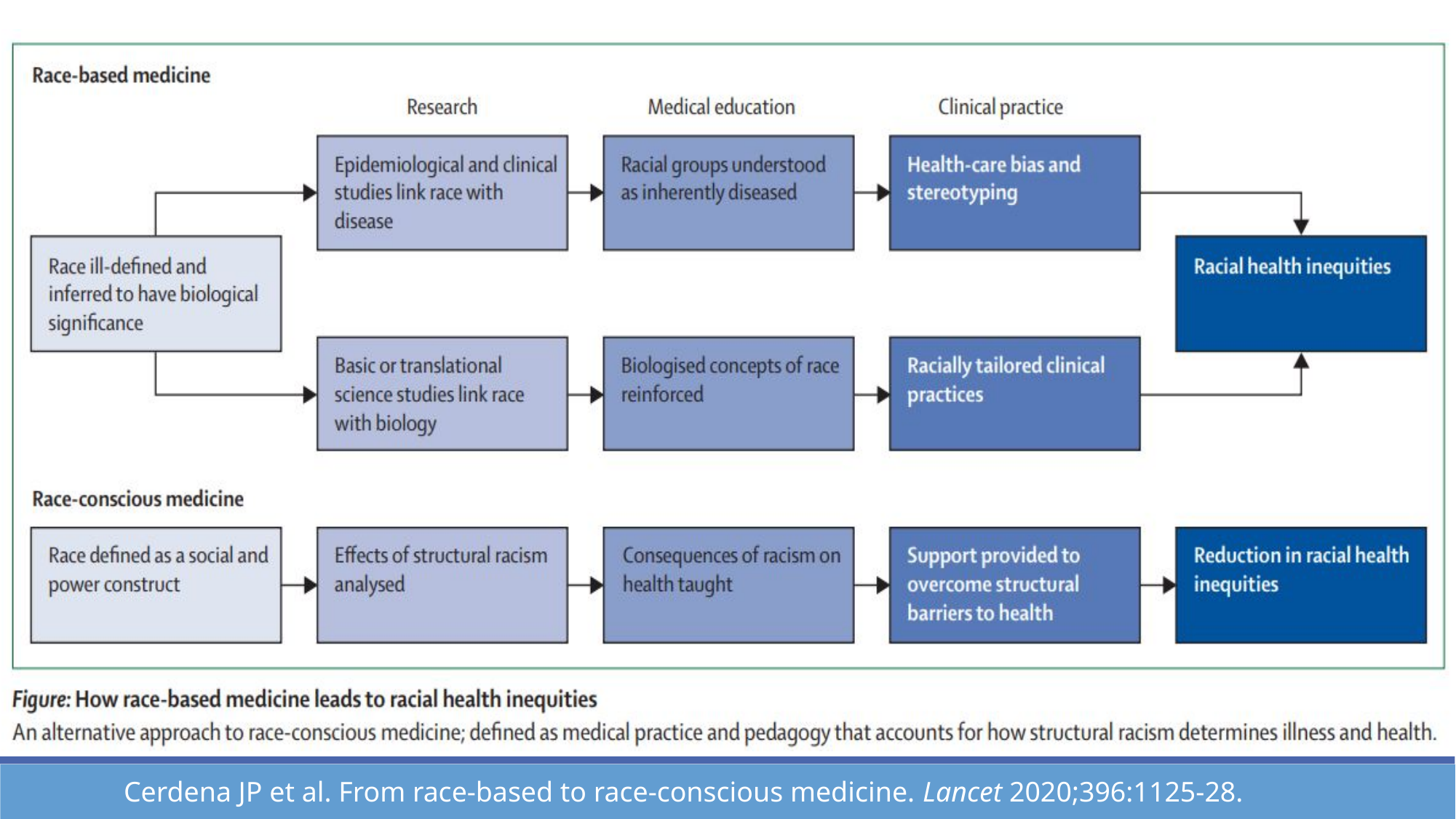

Cerdena JP et al. From race-based to race-conscious medicine. Lancet 2020;396:1125-28.

## Slide 33
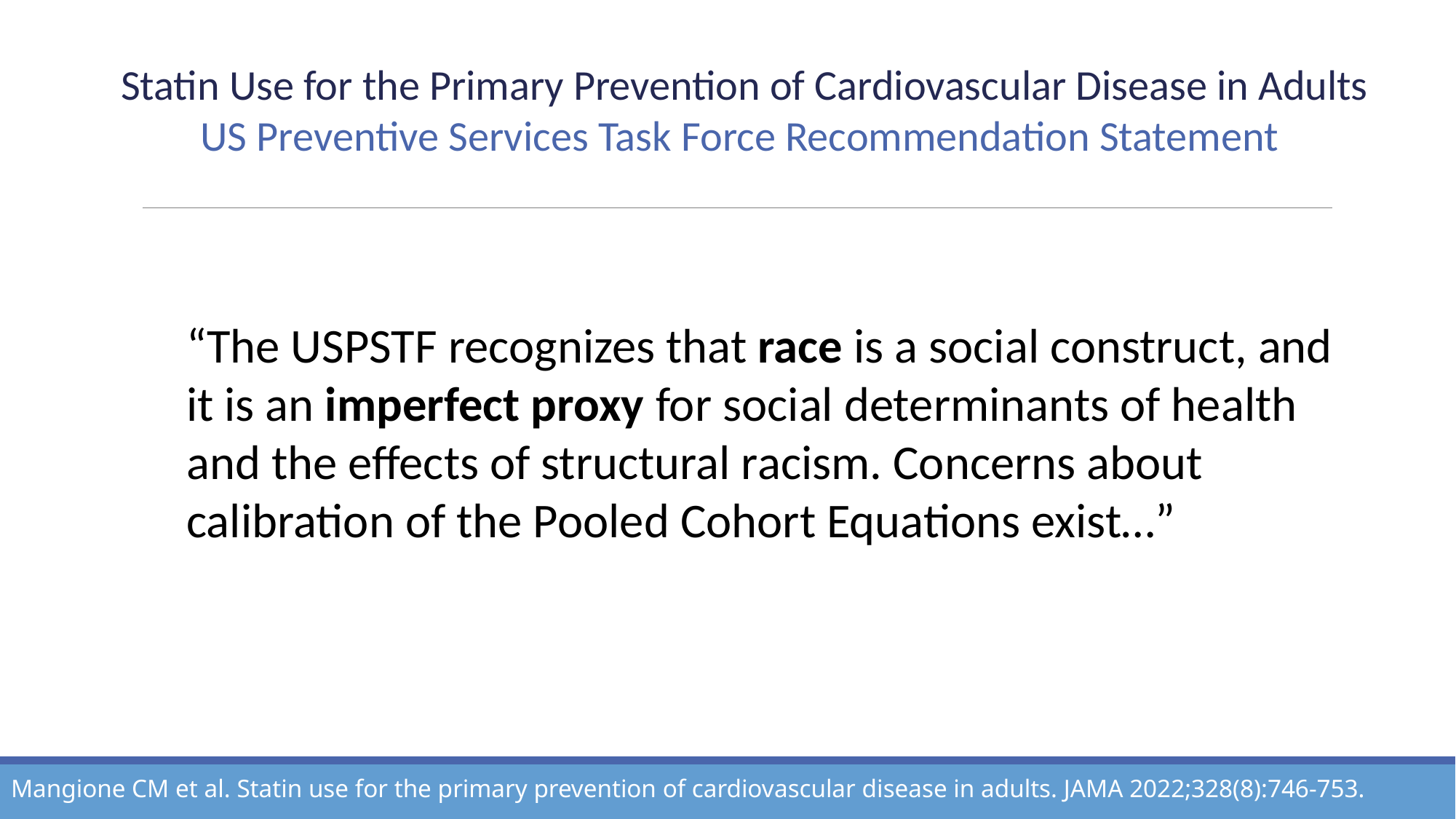

Statin Use for the Primary Prevention of Cardiovascular Disease in Adults
US Preventive Services Task Force Recommendation Statement
“The USPSTF recognizes that race is a social construct, and it is an imperfect proxy for social determinants of health and the effects of structural racism. Concerns about calibration of the Pooled Cohort Equations exist…”
Mangione CM et al. Statin use for the primary prevention of cardiovascular disease in adults. JAMA 2022;328(8):746-753.

## Slide 34
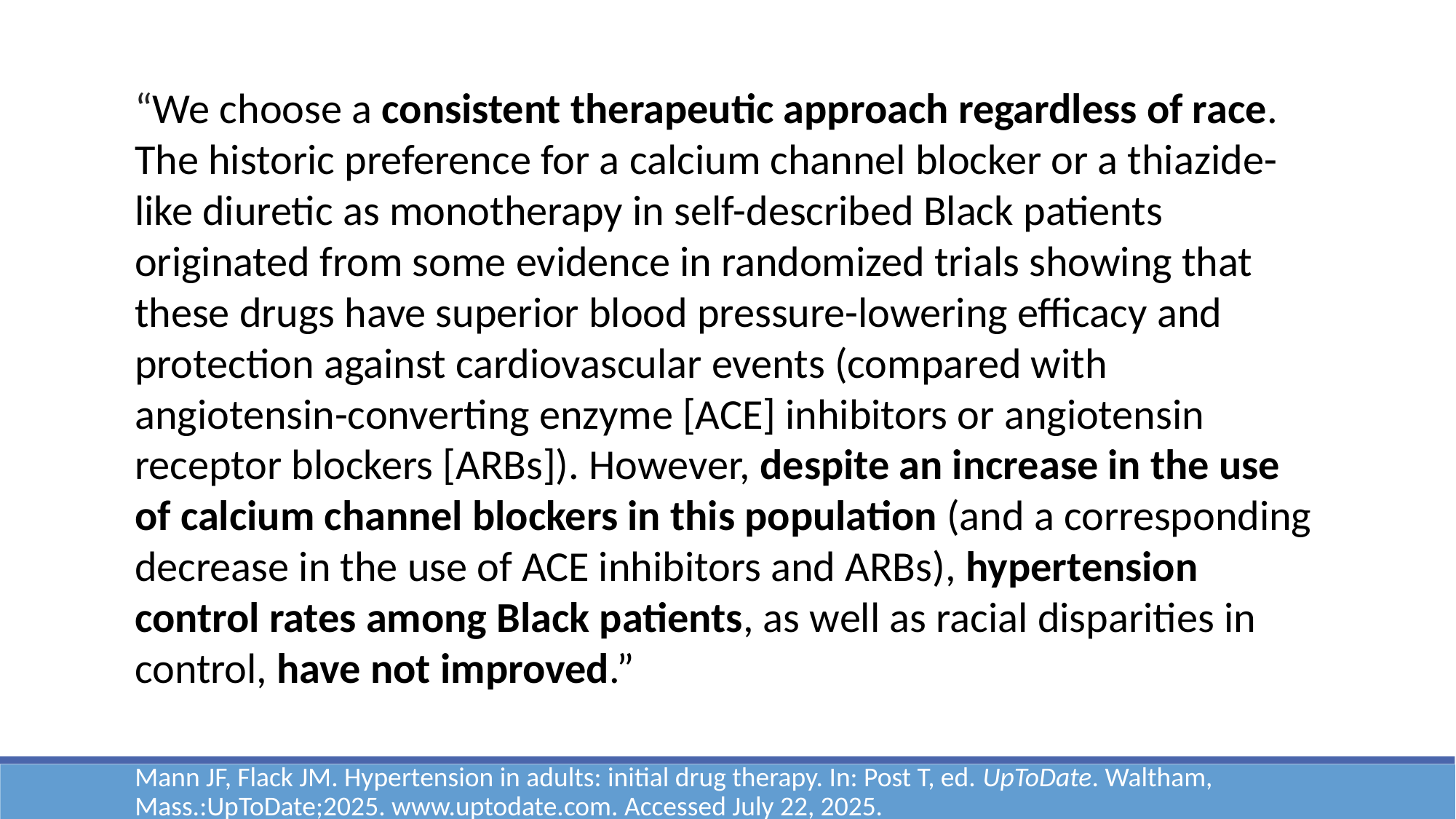

“We choose a consistent therapeutic approach regardless of race. The historic preference for a calcium channel blocker or a thiazide-like diuretic as monotherapy in self-described Black patients originated from some evidence in randomized trials showing that these drugs have superior blood pressure-lowering efficacy and protection against cardiovascular events (compared with angiotensin-converting enzyme [ACE] inhibitors or angiotensin receptor blockers [ARBs]). However, despite an increase in the use of calcium channel blockers in this population (and a corresponding decrease in the use of ACE inhibitors and ARBs), hypertension control rates among Black patients, as well as racial disparities in control, have not improved.”
Mann JF, Flack JM. Hypertension in adults: initial drug therapy. In: Post T, ed. UpToDate. Waltham, Mass.:UpToDate;2025. www.uptodate.com. Accessed July 22, 2025.

## Slide 35
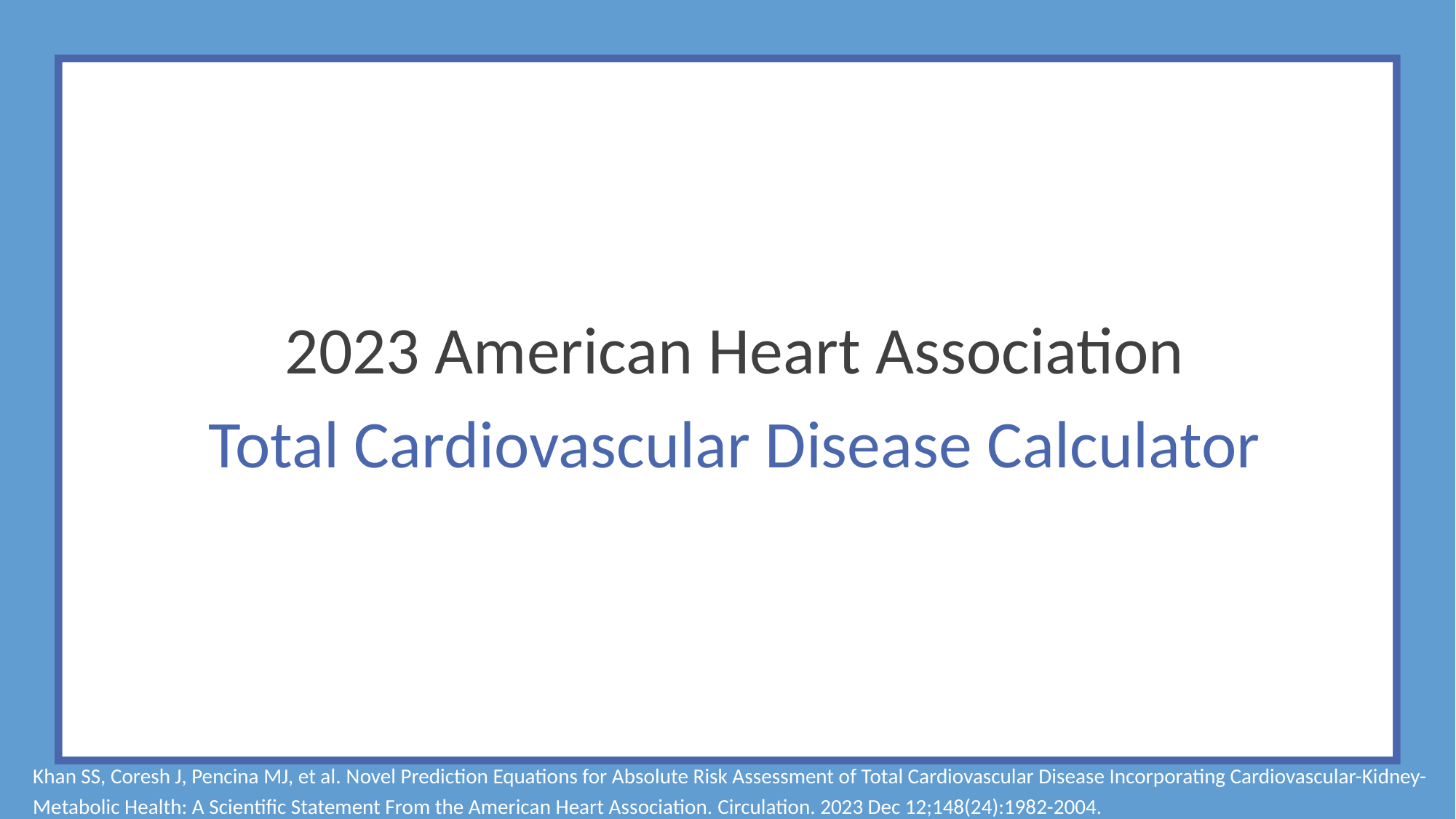

2023 American Heart Association
Total Cardiovascular Disease Calculator
Khan SS, Coresh J, Pencina MJ, et al. Novel Prediction Equations for Absolute Risk Assessment of Total Cardiovascular Disease Incorporating Cardiovascular-Kidney-Metabolic Health: A Scientific Statement From the American Heart Association. Circulation. 2023 Dec 12;148(24):1982-2004.

## Slide 36
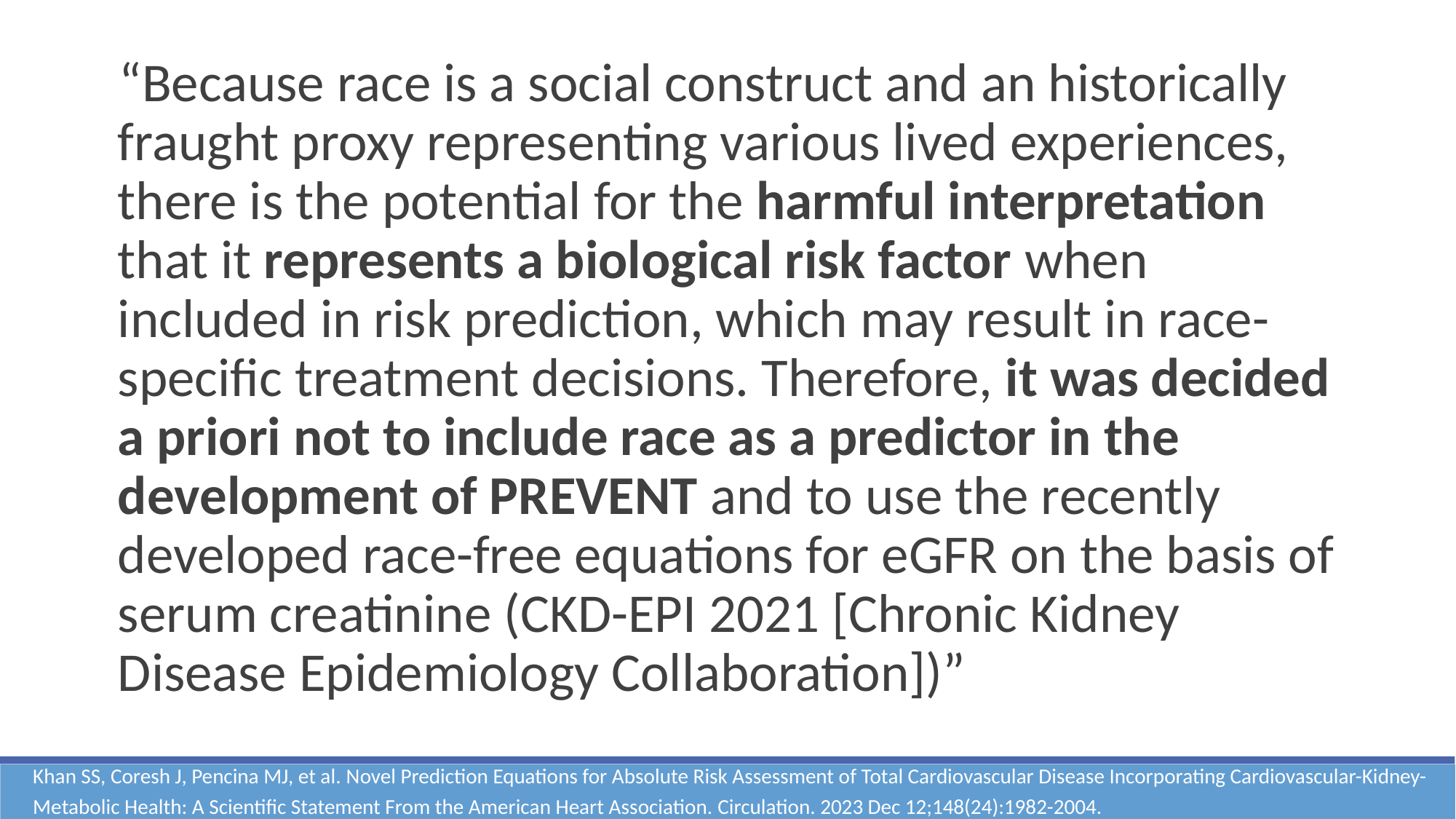

“Because race is a social construct and an historically fraught proxy representing various lived experiences, there is the potential for the harmful interpretation that it represents a biological risk factor when included in risk prediction, which may result in race-specific treatment decisions. Therefore, it was decided a priori not to include race as a predictor in the development of PREVENT and to use the recently developed race-free equations for eGFR on the basis of serum creatinine (CKD-EPI 2021 [Chronic Kidney Disease Epidemiology Collaboration])”
Khan SS, Coresh J, Pencina MJ, et al. Novel Prediction Equations for Absolute Risk Assessment of Total Cardiovascular Disease Incorporating Cardiovascular-Kidney-Metabolic Health: A Scientific Statement From the American Heart Association. Circulation. 2023 Dec 12;148(24):1982-2004.

## Slide 37
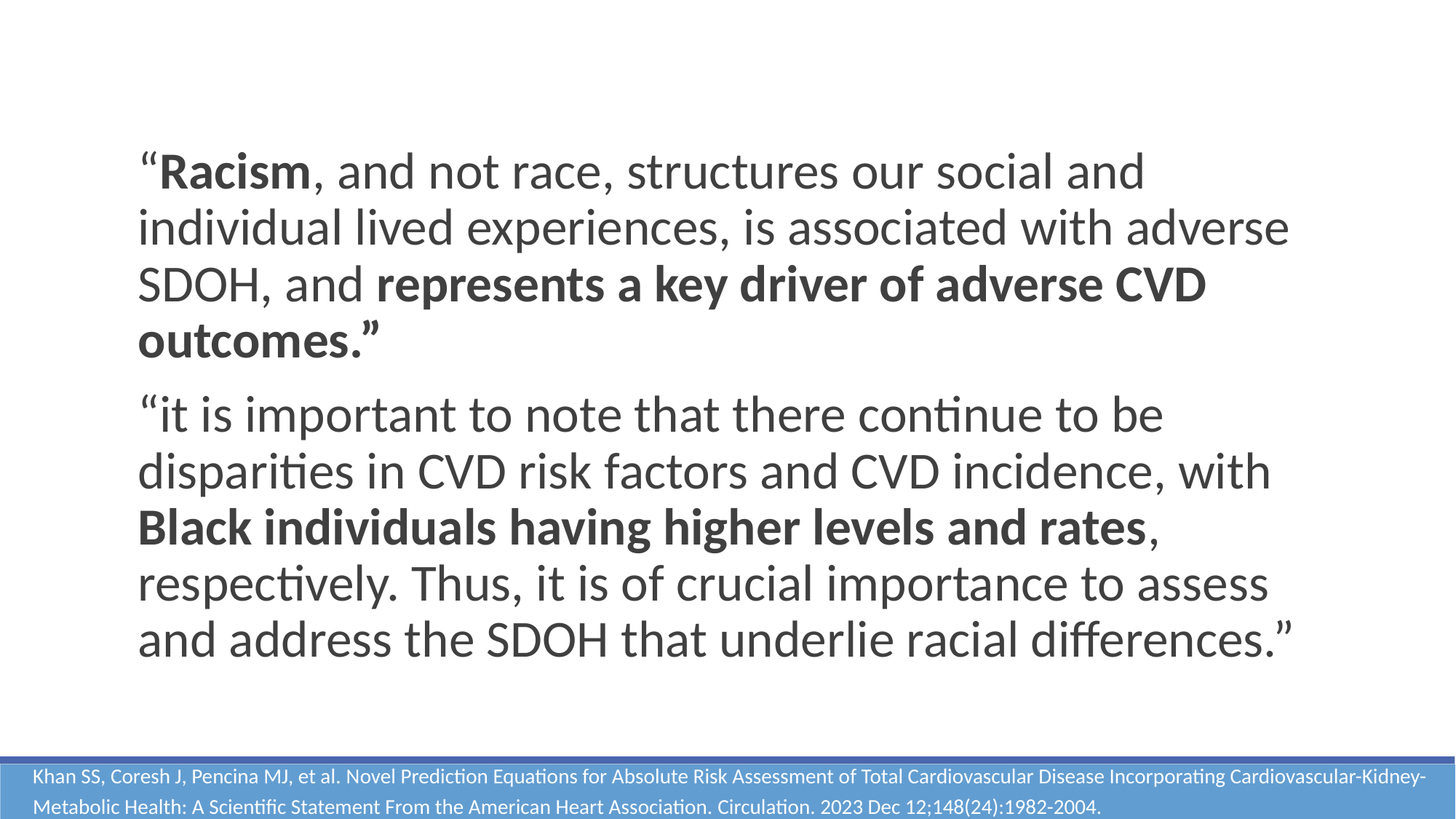

“Racism, and not race, structures our social and individual lived experiences, is associated with adverse SDOH, and represents a key driver of adverse CVD outcomes.”
“it is important to note that there continue to be disparities in CVD risk factors and CVD incidence, with Black individuals having higher levels and rates, respectively. Thus, it is of crucial importance to assess and address the SDOH that underlie racial differences.”
Khan SS, Coresh J, Pencina MJ, et al. Novel Prediction Equations for Absolute Risk Assessment of Total Cardiovascular Disease Incorporating Cardiovascular-Kidney-Metabolic Health: A Scientific Statement From the American Heart Association. Circulation. 2023 Dec 12;148(24):1982-2004.

## Slide 38
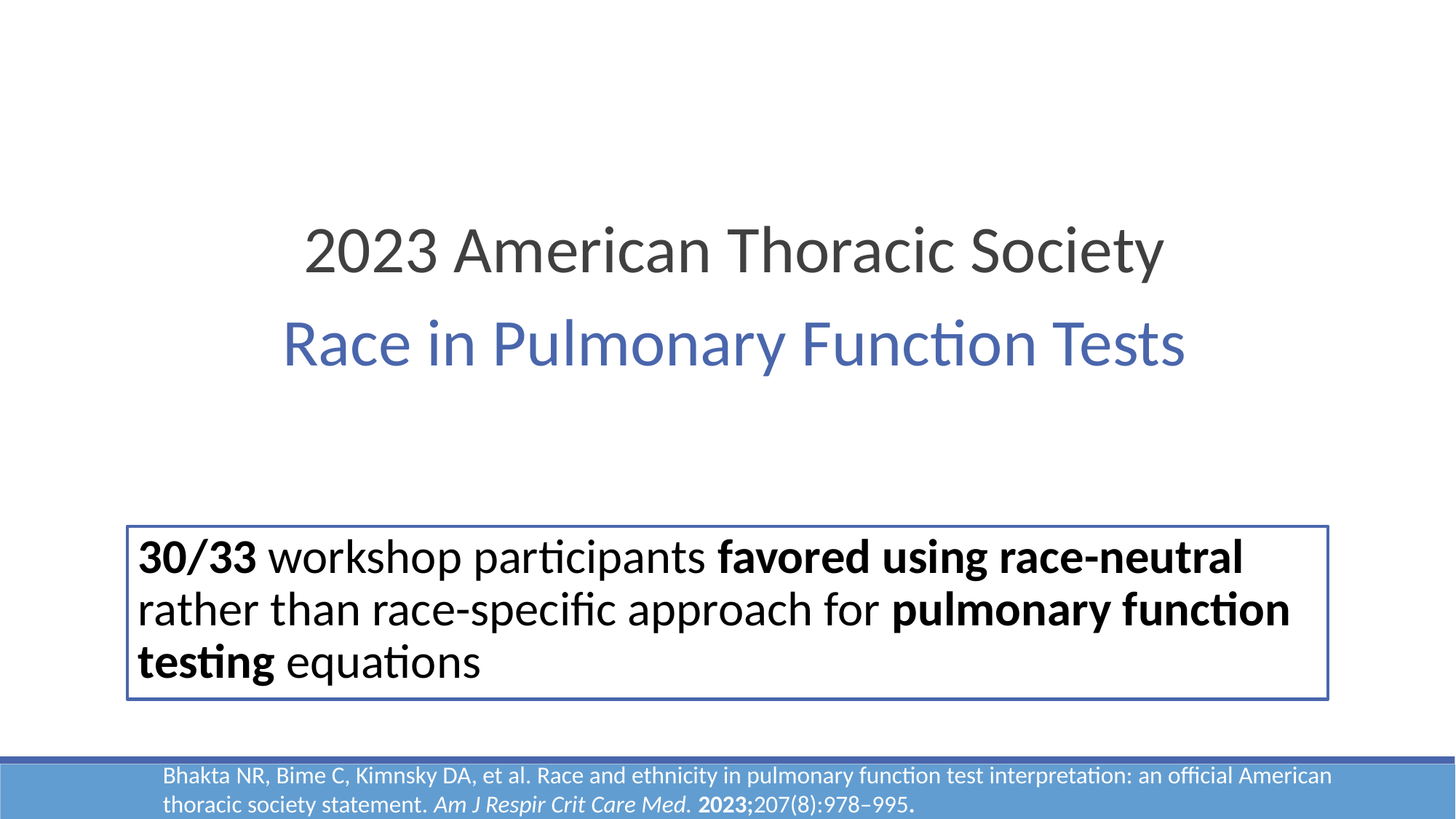

2023 American Thoracic Society
Race in Pulmonary Function Tests
30/33 workshop participants favored using race-neutral rather than race-specific approach for pulmonary function testing equations
Bhakta NR, Bime C, Kimnsky DA, et al. Race and ethnicity in pulmonary function test interpretation: an official American thoracic society statement. Am J Respir Crit Care Med. 2023;207(8):978–995.

## Slide 39
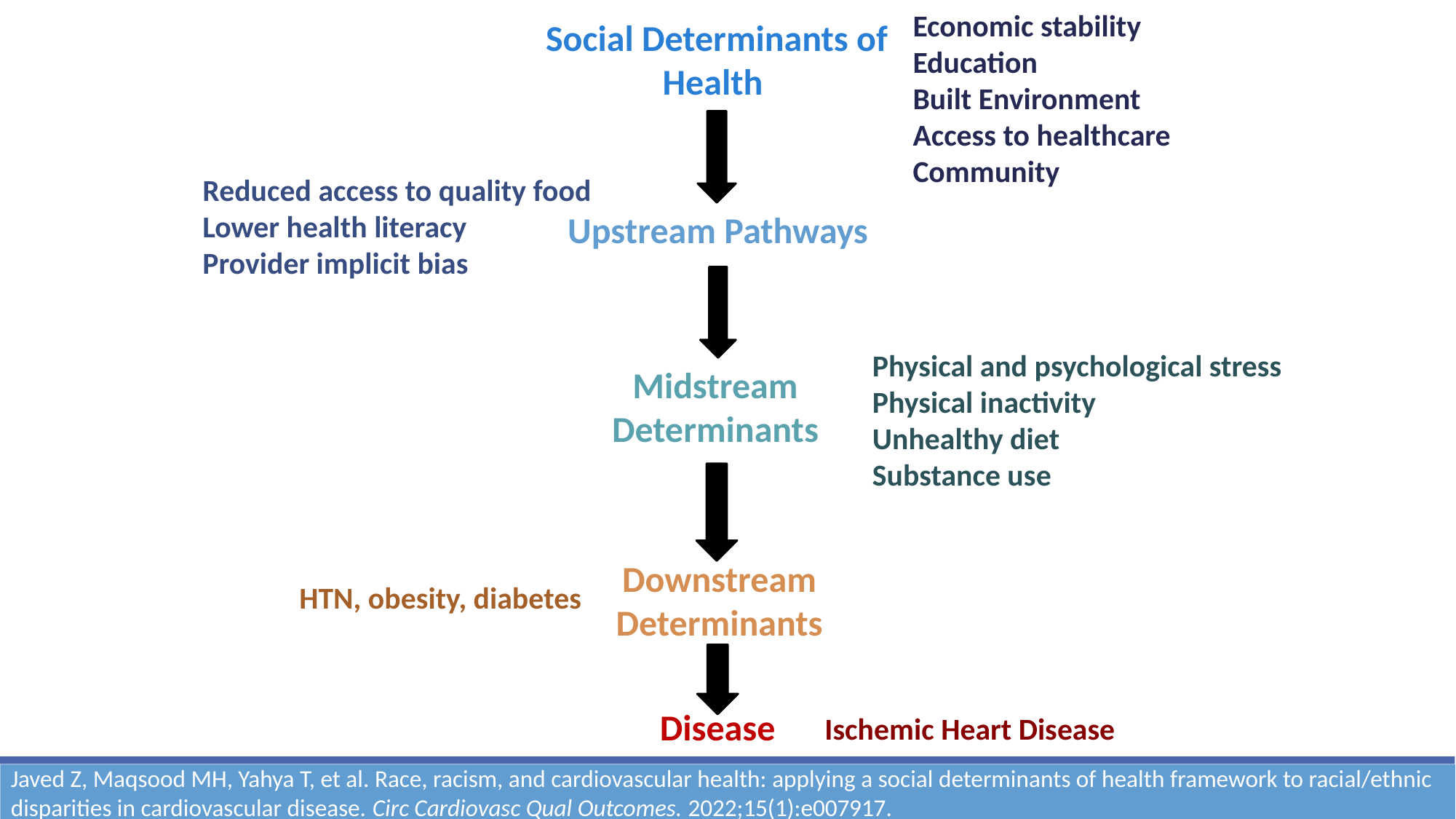

Economic stability
Education
Built Environment
Access to healthcare
Community
Social Determinants of Health
Reduced access to quality food
Lower health literacy
Provider implicit bias
Upstream Pathways
Physical and psychological stress
Physical inactivity
Unhealthy diet
Substance use
Midstream Determinants
Downstream Determinants
HTN, obesity, diabetes
Disease
Ischemic Heart Disease
Javed Z, Maqsood MH, Yahya T, et al. Race, racism, and cardiovascular health: applying a social determinants of health framework to racial/ethnic disparities in cardiovascular disease. Circ Cardiovasc Qual Outcomes. 2022;15(1):e007917.

## Slide 40
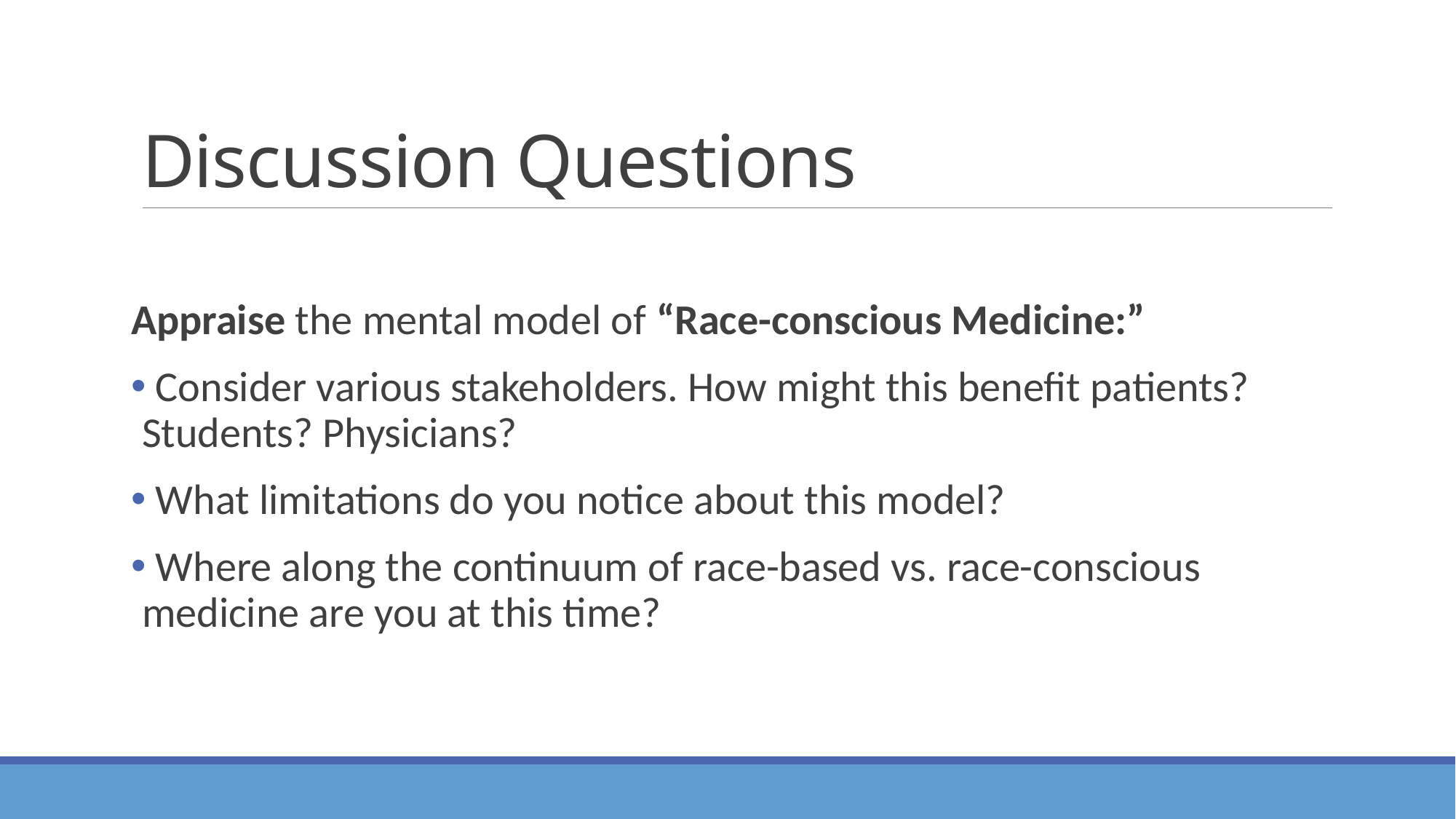

# Discussion Questions
Appraise the mental model of “Race-conscious Medicine:”
 Consider various stakeholders. How might this benefit patients? Students? Physicians?
 What limitations do you notice about this model?
 Where along the continuum of race-based vs. race-conscious medicine are you at this time?

## Slide 41
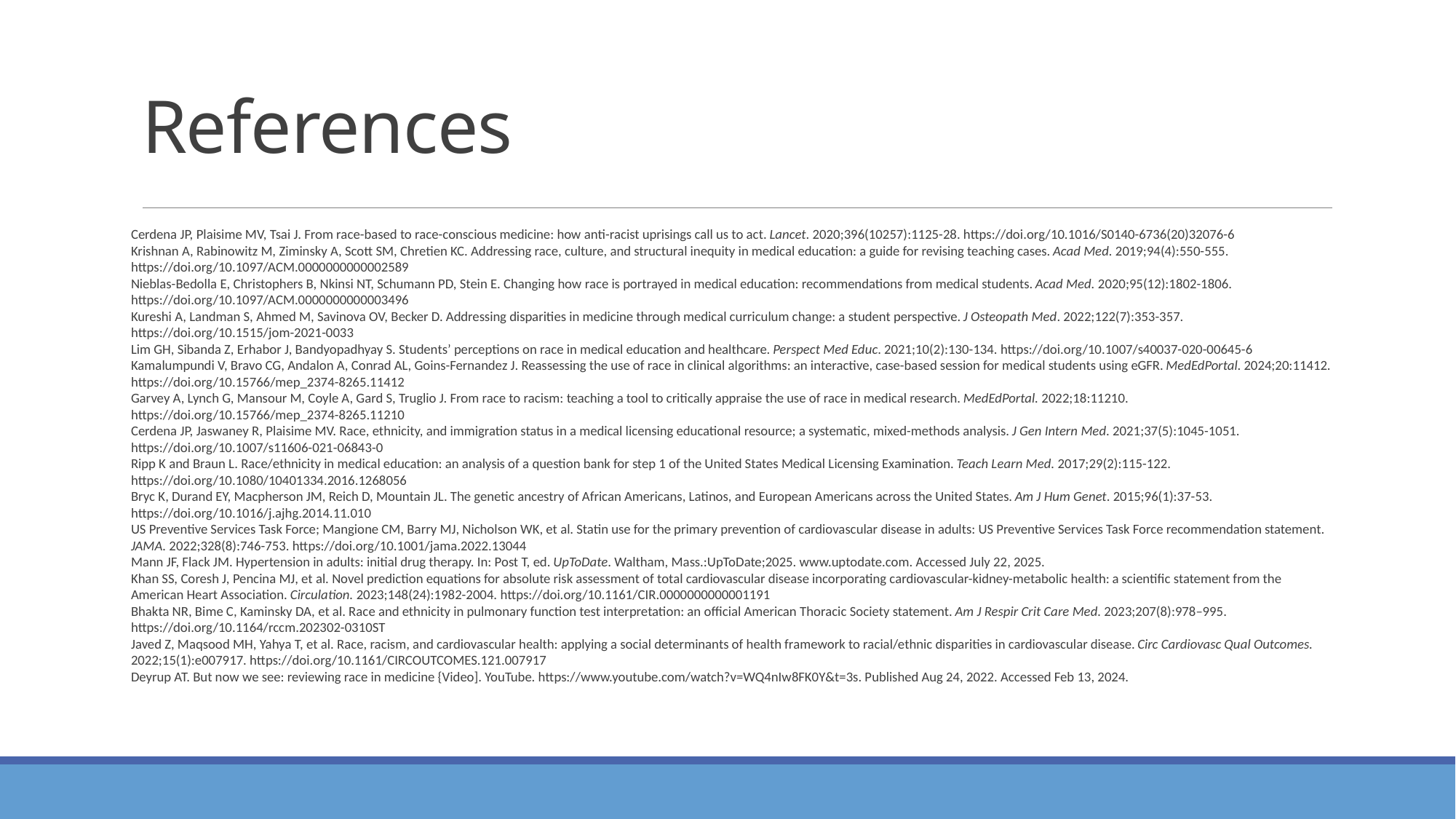

# References
Cerdena JP, Plaisime MV, Tsai J. From race-based to race-conscious medicine: how anti-racist uprisings call us to act. Lancet. 2020;396(10257):1125-28. https://doi.org/10.1016/S0140-6736(20)32076-6
Krishnan A, Rabinowitz M, Ziminsky A, Scott SM, Chretien KC. Addressing race, culture, and structural inequity in medical education: a guide for revising teaching cases. Acad Med. 2019;94(4):550-555. https://doi.org/10.1097/ACM.0000000000002589
Nieblas-Bedolla E, Christophers B, Nkinsi NT, Schumann PD, Stein E. Changing how race is portrayed in medical education: recommendations from medical students. Acad Med. 2020;95(12):1802-1806. https://doi.org/10.1097/ACM.0000000000003496
Kureshi A, Landman S, Ahmed M, Savinova OV, Becker D. Addressing disparities in medicine through medical curriculum change: a student perspective. J Osteopath Med. 2022;122(7):353-357. https://doi.org/10.1515/jom-2021-0033
Lim GH, Sibanda Z, Erhabor J, Bandyopadhyay S. Students’ perceptions on race in medical education and healthcare. Perspect Med Educ. 2021;10(2):130-134. https://doi.org/10.1007/s40037-020-00645-6
Kamalumpundi V, Bravo CG, Andalon A, Conrad AL, Goins-Fernandez J. Reassessing the use of race in clinical algorithms: an interactive, case-based session for medical students using eGFR. MedEdPortal. 2024;20:11412. https://doi.org/10.15766/mep_2374-8265.11412
Garvey A, Lynch G, Mansour M, Coyle A, Gard S, Truglio J. From race to racism: teaching a tool to critically appraise the use of race in medical research. MedEdPortal. 2022;18:11210. https://doi.org/10.15766/mep_2374-8265.11210
Cerdena JP, Jaswaney R, Plaisime MV. Race, ethnicity, and immigration status in a medical licensing educational resource; a systematic, mixed-methods analysis. J Gen Intern Med. 2021;37(5):1045-1051. https://doi.org/10.1007/s11606-021-06843-0
Ripp K and Braun L. Race/ethnicity in medical education: an analysis of a question bank for step 1 of the United States Medical Licensing Examination. Teach Learn Med. 2017;29(2):115-122. https://doi.org/10.1080/10401334.2016.1268056
Bryc K, Durand EY, Macpherson JM, Reich D, Mountain JL. The genetic ancestry of African Americans, Latinos, and European Americans across the United States. Am J Hum Genet. 2015;96(1):37-53. https://doi.org/10.1016/j.ajhg.2014.11.010
US Preventive Services Task Force; Mangione CM, Barry MJ, Nicholson WK, et al. Statin use for the primary prevention of cardiovascular disease in adults: US Preventive Services Task Force recommendation statement. JAMA. 2022;328(8):746-753. https://doi.org/10.1001/jama.2022.13044
Mann JF, Flack JM. Hypertension in adults: initial drug therapy. In: Post T, ed. UpToDate. Waltham, Mass.:UpToDate;2025. www.uptodate.com. Accessed July 22, 2025.
Khan SS, Coresh J, Pencina MJ, et al. Novel prediction equations for absolute risk assessment of total cardiovascular disease incorporating cardiovascular-kidney-metabolic health: a scientific statement from the American Heart Association. Circulation. 2023;148(24):1982-2004. https://doi.org/10.1161/CIR.0000000000001191
Bhakta NR, Bime C, Kaminsky DA, et al. Race and ethnicity in pulmonary function test interpretation: an official American Thoracic Society statement. Am J Respir Crit Care Med. 2023;207(8):978–995. https://doi.org/10.1164/rccm.202302-0310ST
Javed Z, Maqsood MH, Yahya T, et al. Race, racism, and cardiovascular health: applying a social determinants of health framework to racial/ethnic disparities in cardiovascular disease. Circ Cardiovasc Qual Outcomes. 2022;15(1):e007917. https://doi.org/10.1161/CIRCOUTCOMES.121.007917
Deyrup AT. But now we see: reviewing race in medicine {Video]. YouTube. https://www.youtube.com/watch?v=WQ4nIw8FK0Y&t=3s. Published Aug 24, 2022. Accessed Feb 13, 2024.

## Slide 42
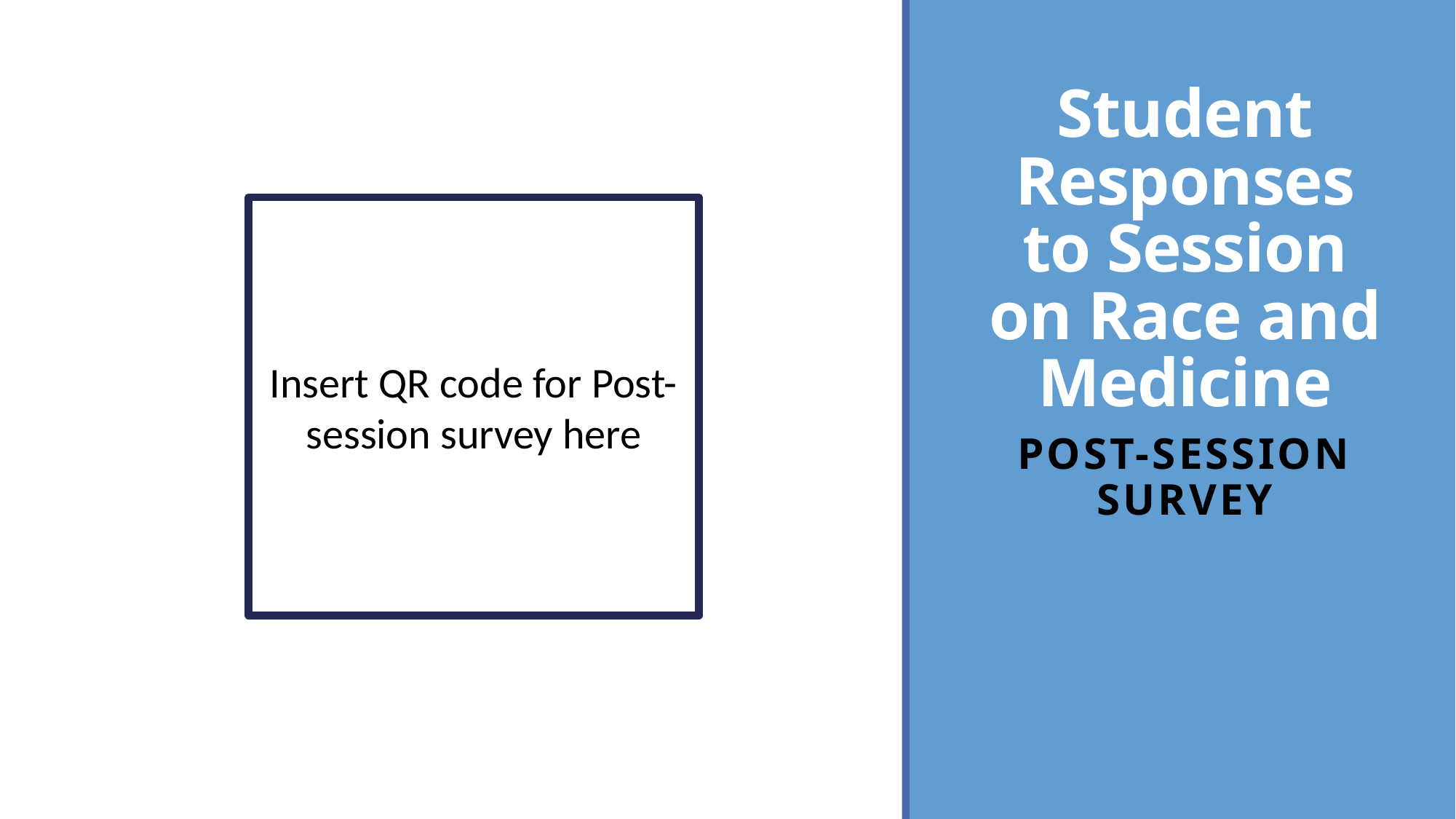

# Student Responses to Session on Race and Medicine
Insert QR code for Post-session survey here
post-Session Survey
