# Supplementary material for: Exploring the Complexity of Considering Race in the Practice of Medicine
Source: MedEdPORTAL. 2026 Mar 17;22:11585. doi: 10.15766/mep_2374-8265.11585 (PMC12992537; doi:10.15766/mep_2374-8265.11585)
Supplement: Supplementary file 1 — Race and Medicine.pptxFacilitator Guide.docxPre- and Postsession Survey.docx [file mep_2374-8265.11585-s001.zip › B. Facilitator Guide.docx]

**Race and Medicine**

Facilitator Guide

**Session Overview:** This session is designed to present and critique the traditional race-based approach to considering race in medicine. The underlying framework of race-based medicine is that race is a proxy for genetic or biological influences on health. We survey examples of how this framework is applied in medical education and clinical settings, encouraging students to rethink this approach to race and medicine. This session will conclude with an alternative approach—race-conscious medicine—which emphasizes the structural and social impacts of race in health care.

**Learning Objectives:**

1. Recognize the ways race is used as a proxy for genetic determinants of health.
2. Evaluate the use of race as an indicator of disease risk or response to therapy.
3. Assess the risks and benefits of the ongoing practice of race-based medicine.
4. Describe evolving frameworks for considering race in medicine.

**Pre-session Reading:** Ask students to read the following article before arriving to class:

Cerdena JP, Plaisime MV, Tsai J. From race-based to race-conscious medicine: how anti-racist uprisings call us to act. *Lancet* 2020;396(10257):1125-1128

**Classroom Setup:** Students will be engaging in small group discussion at several points during the presentation. Encourage learners to sit within groups of 4-8 students to facilitate transitions to small group discussion. Encourage faculty to sit among student groups at the beginning of the session. While we utilized 12 faculty, this number can be scaled depending on class size and available faculty. If there are fewer faculty than student groups, each faculty could alternate between two student groups.

**Faculty Preparation:**

- Glossary of terms (from above article by Cerdena et al)
  - Race-based medicine: framework that characterizes race as an essential biological variable for research and clinical practice
  - Race-conscious medicine: framework that emphasizes structural and social determinants of health as the primary drivers for race-related health disparities
- Facilitation instructions:
  - Faculty should read the assigned article and review discussion questions prior to class
  - Faculty should be briefed on the importance of psychological safety when discussing these questions
  - Faculty should promote reflection by asking open-ended follow-up questions to facilitate students to share varying perspectives. They should avoid sharing their own opinions or leaning into their content expertise during discussions.
  - It is expected that students will not arrive at conclusive discussion points or “correct” answers
  - Consider this format when facilitating discussion: (1) ask a question on the presentation slide, (2) listen to students’ responses, (3) ask the group about additional perspectives, (4) if applicable, share your own curiosity or areas of uncertainty to model intellectual humility

**Preparation Materials:** Open up the following on your internet browser prior to beginning the session:

- Audience response tool (e.g. Slido.com). Slido is available for free with a maximum of 3 questions. I created the following word cloud questions:
  - Question 1: What are your thoughts about using this definition of race in medicine? (see slide 6)
  - Question 2: What diagnoses are you considering? (see slide 8)
  - Question 3: What diagnoses are you considering? (see slide 9)
- American College of Cardiology Atherosclerotic Cardiovascular Disease (ASCVD) Risk Estimator Plus
- Web-based timer to visually indicate remaining duration for small group discussion

**Session Format and Timeline**: approximately 90 minutes total.

| **Section** | **Slide number(s)** | **Time** |
| --- | --- | --- |
| Pre-session survey | 2 | 3 minutes |
| Race used as proxy for genetic determinants of health | 4-12 | Didactic: 15 minutes  Discussion: 5 minutes |
| Questioning association of race with disease risk and response to therapy | 13-17 | Didactic: 5 min  Discussion: 10 minutes |
| Summarizing and appraising the use of race-based medicine in cardiovascular medicine | 18-29 | Didactic 15 minutes  Case discussion: 10 minutes* |
| Exploring other frameworks for race in medicine | 30-40 | Didactic: 15 minutes  Discussion: 10 minutes |
| Post-session survey | 42 | 3 minutes |

*If feasible, this case discussion could benefit from 3-5 minutes of additional discussion time.

**Slide Instructions**

Slide 1: Title slide

Slide 2: Insert QR code to pre-session survey

Slide 3: Session learning outcomes. The key elements to highlight are that this presentation will:

- Present ways race is used, either implicitly or explicitly, as a proxy for genetic and biological determinants of health
- Give students the opportunity to rethink this approach, weighing the benefits and harms of this framework
- Discuss alternate ways to consider race in medicine

Slide 4: Section heading to introduce presentation of first learning objective

Slide 5: This slide provides a starting definition of race, at least within the United States. As you present this slide, consider asking the following questions for large group discussion:

- What are your thoughts about this definition?
- Consider who is left out. Notice the diversity of race represented in this room.
- What might this mean for medicine?

Slide 6: Audience response tool on above definition of race in medicine

- Open up browser with first audience response question: “What are your thoughts about using this definition of race in medicine?” Note: the definition referred to here is that of the US Census Bureau.
- At this point, certain audience response tools (e.g., slido) may require you to begin the session on the platform website. It can be helpful to have this open on another browser in advance. You can then slide this over to the presentation screen so students can view peer responses.
- This is an opportunity for those who are not comfortable sharing out loud to contribute to this discussion. Consider reading some of the responses out loud.

Slide 7: Examples of race-based medicine presented in article

Slide 8-9: Engaged demonstration of how race might influence students’ differential diagnoses

- Slide 8 doesn’t include race. Students might consider a variety of diagnoses (e.g. rosacea, fibromyalgia, systemic lupus erythematosus).
- Slide 9 adds African American race. The intention is to see if more students respond with systemic lupus erythematosus (SLE) when African American race is included.
- Even if a significant change in responses is not seen, it could be helpful to ask students to reflect on whether adding race strengthened the confidence in their diagnosis of SLE

Slide 10 and 11: Race used as part of pattern-recognition in standardized testing

- Slide 10: In this study, race was central to the diagnosis far more often when a racial minority was included in the question stem
- Slide 11: In this study, black race was disproportionately included in question stems. This could have various impacts, including:
  - Seeing black people as more likely to develop disease in general
  - Association of certain diseases with black race

Slide 12: Small Group Discussion. Ask students to break up into small groups to discuss the listed questions. I recommend not wrapping this session up with correct answers but leaving room for ambivalence as they continue to engage in the remainder of the presentation. It may be helpful to display a web-based timer so students are able to gauge the remaining time. This facilitated their transition back to the didactic presentation when discussion time ended.

Slide 13: Transition slide to highlight second learning objective

Slide 14: Return to the examples of race-based medicine from slide 7. This is a structured opportunity for students to critically analyze the framework behind the examples cited. Based on timing and group dynamics, this can be done as a large group discussion or think-pair-share. If time permits, ask students to review the table in the pre-reading article to see the rationale cited for each example of race-based medicine.

Slides 15-16: Address geographic ancestry as an argument in favor of race-based medicine.

- The argument is that people of different races carry similar genes due to their geographic ancestry.
- The problem with this argument, as the paper by Byrc et al. highlights, is that self-identified race doesn’t have a clear association with geographic ancestry

Slide 17: Small Group Discussion. Ask students to break up into small groups to discuss the listed questions. A visual timer is again recommended. Again, I recommend leaving this discussion open-ended, without presenting the “correct answers” to students.

Slide 18: Transition slide to highlight third learning objective.

Slide 19: This section is going to focus specifically on race and cardiovascular disease

- This system was chosen due to the common and widespread use of race-based tools in cardiovascular medicine
- This session should ideally be presented after students have learned about the basics of the cardiovascular system

Slide 20: Present a well-renown article that summarizes current recommendations on management of hyperlipidemia.

Slide 21: Pull up the American College of Cardiology Atherosclerotic Cardiovascular Disease (ASCVD) risk estimator:

- American College of Cardiology. ASCVD risk estimator plus. Accessed August 15, 2025. https://tools.acc.org/ascvd-risk-estimator-plus/#!/calculate/estimate
- Use the following data to show how the estimated risk is significantly different for White vs. African American women: age 64, SBP 135/80, Total 220, HDL 38, LDL 130
- Change “sex” to male to see how that estimator not only varies by race but also by sex

Slides 22-23: Reference 2017 AHA hypertension guidelines, another frequently used guideline for managing cardiovascular disease

- Highlight the difference of initial-line therapy options based on race
- Highlight the stronger recommendation for multi-drug therapy for Black patient

Slides 24-25: Reference 2022 American Heart Association guidelines for heart failure management

- Highlight the nuanced recommendation of hydralazine and nitrates for Black patients
- This could lead to polypharmacy and/or decreased consistency of medication use

Slides 26-27: This case scenario was designed to highlight management differences between White and Black patients with hypertension and hyperlipidemia

- Ask students to access the ACC risk estimator calculator on their laptops
- Give students 3-5 minutes to read the case, utilize the calculator, and notice how a strict adherence to race-based recommendations would change their care for this hypothetical patient

Slide 28: Return to the examples of race-based medicine discussed earlier in the presentation (slide 7).

- This discussion can occur as a large group, with students sharing their reflections with the entire class.
- This could also occur in the same small groups as the previous activity (slide 27)
- Refer to table from article by Cerdena et al. for rationale and potential harm.

Slide 29: Discussion Questions. This slide is included to promote both/and thinking on this topic. While a formulaic approach to race and medicine (“race-based medicine”) can lead to potential harm, perhaps we should still consider race in medicine. Perhaps there are other frameworks, other than genetic ancestry, for considering race and medicine.

- This slide could be shared as a brief rhetorical set of questions for reflection
- It could also be presented as an opportunity for brief large-group discussion

Slide 30: Transition slide to 4^th^ learning outcome

Slide 31: Reflection on the framework connecting race and medicine

- Recapping the biological framework of “race-based medicine”
- Considering an updated framework involving a structural racism paradigm or the impact of social determinants of health

Slide 32: Slide from article by Cerdena et al. that proposes the impacts of a “race-conscious” framework on research, medical education, and clinical practice

- Race-based medicine’s linkage of race with biology can reinforce health care biases and stereotypes and worsen health care inequities
- Race-conscious medicine’s recognition of structural racism can prompt intentional work to address structural barriers and thus reduce health care disparities

Slide 33: Return to the USPSTF article on the use of statins in primary prevention of cardiovascular disease, referenced in slide 20

- This quote highlights the authors’ acknowledgement of some doubt with the Pooled Cohort Equations used to develop the race-based component of the ACC Risk estimator calculator.
- This can provide support to students worried about the medical-legal implications of not using race in this risk calculator

Slide 34: Excerpt from a 2025 UpToDate article that proposes an alternative approach to choosing a first-line medication for hypertension.

- This highlights that expert opinion is varied on this topic
- Again, this provides support for students concerned about not following race-based recommendations of the 2017 AHA guidelines for hypertension

Slides 35-37: Introduce the scientific statement article on the AHA “PREVENT” Calculator, which estimates the composite risk of ASCVD and heart failure. This calculator does not include race. It does, however, include zip code to estimate “social deprivation index”

- This is a powerful example of how experts in the field are creating updated tools founded on a social determinant of health model instead of a race-based framework
- Link to PREVENT online calculator:
  - American Heart Association. The American Heart Association PREVENT online calculator. Accessed August 15, 2025. https://professional.heart.org/en/guidelines-and-statements/prevent-risk-calculator/prevent-calculator
  - Can pull up he calculator to highlight that it doesn’t include race
- Slides 36-37 is include excerpts from the scientific statement article that explains the authors’ intentional exclusion of race in the calculator

Slide 38: Another official statement, this time related to the use of race in PFT interpretation, supporting the exclusion of race in this calculator

Slide 39: Flow chart of a portion of the referenced article by Javid et al. highlighting an updated framework for how race can be one factor that impacts health outcomes

- In this flow chart, the key factors under consideration are social determinants of health, rather than race or ethnicity alone
- This framework considers a breadth of factors that can help explain some disparities in disease outcomes associated with various races or ethnicities
- This framework encourages inquiry into the impact of social determinants of health (SDOH) on cardiovascular disease, an area of ongoing discovery that has been long neglected because of the assumption of race-based biological differences in disease and treatment responses

Slide 40: Final set of discussion questions to reflect on the “race-conscious” or structural and social determinant framework of considering race in medicine

- These questions can be discussed in small groups
- The first question asks students to engage in perspective-taking of various stakeholders
- The second question promotes intellectual curiosity and critical thinking
- The third question encourages a nuanced, rather than either/or, consideration of race and medicine.

Slide 41: Reference slide

Slide 42: Post-session survey
