# Supplementary material for: Exploring the Complexity of Considering Race in the Practice of Medicine
Source: MedEdPORTAL. 2026 Mar 17;22:11585. doi: 10.15766/mep_2374-8265.11585 (PMC12992537; doi:10.15766/mep_2374-8265.11585)
Supplement: Supplementary file 1 — Race and Medicine.pptxFacilitator Guide.docxPre- and Postsession Survey.docx [file mep_2374-8265.11585-s001.zip › C. Pre- and Postsession Survey.docx]

**Pre- and Postsession Survey**

**Title:** M1 Student Responses to a Session on Race and Medicine

**Instructions:** This same survey can be disseminated to students both before and after the session. An IRB-approved consent form can be included as an image at the beginning of the survey. The first survey question can ask for informed consent for participation in the study (yes/no).

Please select ONE of the following responses for each question:

|  | Strongly Disagree | Disagree | Neutral | Agree | Strongly Agree |
| --- | --- | --- | --- | --- | --- |
| 1. Race is a helpful category for approximating genetic-associated risk of disease. | [ ] | [ ] | [ ] | [ ] | [ ] |
| 1. Race is a social construct that is a poor proxy for determining biological differences among people groups. | [ ] | [ ] | [ ] | [ ] | [ ] |
| 1. It is more beneficial than harmful to consider a patient’s race in the DIAGNOSIS of disease. | [ ] | [ ] | [ ] | [ ] | [ ] |
| 1. It is more beneficial than harmful to consider a patient’s race when developing a THERAPEUTIC PLAN for disease. | [ ] | [ ] | [ ] | [ ] | [ ] |
| 1. It is more beneficial than harmful to consider a patient’s race when conducting MEDICAL RESEARCH. | [ ] | [ ] | [ ] | [ ] | [ ] |
| 1. A patient's race should be considered MORE from the lens of structural and social determinants of health RATHER THAN biological differences among people groups. | [ ] | [ ] | [ ] | [ ] | [ ] |
| 1. It is more beneficial than harmful to include race when calculating eGFR (estimated glomerular filtration rate). | [ ] | [ ] | [ ] | [ ] | [ ] |
| 1. It is more beneficial than harmful to include race when calculating ASCVD (atherosclerotic cardiovascular disease) risk. | [ ] | [ ] | [ ] | [ ] | [ ] |
| 1. It is more beneficial than harmful to include race in selecting the best choice of therapy for cardiovascular disease (e.g. hypertension or heart failure). | [ ] | [ ] | [ ] | [ ] | [ ] |
| 1. I feel CONFIDENT about my perspective on the relative benefits and risks of considering race in patient care. | [ ] | [ ] | [ ] | [ ] | [ ] |
